# ARNAX is an ideal adjuvant for COVID-19 vaccines to enhance antigen-specific CD4^+^ and CD8^+^ T-cell responses and neutralizing antibody induction

**DOI:** 10.1128/jvi.02290-24

**Published:** 2025-04-15

**Authors:** Tomomi Kawakita, Toshiki Sekiya, Yayoi Kameda, Naoki Nomura, Marumi Ohno, Chimuka Handabile, Akari Yamaya, Hideo Fukuhara, Yuki Anraku, Shunsuke Kita, Shinsuke Toba, Hirotake Tsukamoto, Tomohiro Sawa, Hiroyuki Oshiumi, Yasushi Itoh, Katsumi Maenaka, Akihiko Sato, Hirofumi Sawa, Yasuhiko Suzuki, Lorena E. Brown, David C. Jackson, Hiroshi Kida, Misako Matsumoto, Tsukasa Seya, Masashi Shingai

**Affiliations:** 1 Division of Vaccine Immunology, International Institute for Zoonosis Control, Hokkaido University 12810, Sapporo, Japan; 2 Institute for Vaccine Research and Development (HU-IVReD), Hokkaido University 12810, Sapporo, Japan; 3 Division of Biologics Development, International Institute for Zoonosis Control, Hokkaido University 12810, Sapporo, Japan; 4 International Collaboration Unit, International Institute for Zoonosis Control, Hokkaido University 12810, Sapporo, Japan; 5 The Department of Microbiology and Immunology, The University of Melbourne at the Peter Doherty Institute for Infection and Immunity 2281, Melbourne, Australia; 6 Division of Bioresources, International Institute for Zoonosis Control, Hokkaido University 12810, Sapporo, Japan; 7 Division of International Research Promotion, International Institute for Zoonosis Control, Hokkaido University 12810, Sapporo, Japan; 8 One Health Research Center, Hokkaido University 12810, Sapporo, Japan; 9 Nebuta Research Institute for Life Sciences, Aomori University 12716, Aomori, Japan; 10 Division of Pathogen Structure, International Institute for Zoonosis Control, Hokkaido University 12810, Sapporo, Japan; 11 Laboratory of Biomolecular Science, and Center for Research and Education on Drug Discovery, Faculty of Pharmaceutical Sciences, Hokkaido University 57910 , Sapporo, Japan; 12 Shionogi Pharmaceutical Research Center, Shionogi & Company, Limited, Toyonaka, Japan; 13 Division of Molecular Pathobiology, International Institute for Zoonosis Control, Hokkaido University 12810, Sapporo, Japan; 14 Division of Clinical Immunology and Cancer Immunotherapy, Center for Cancer Immunotherapy and Immunobiology, Graduate School of Medicine, Kyoto University 12918, Kyoto, Japan; 15 Department of Microbiology, Graduate School of Medical Sciences, Kumamoto University 13205, Kumamoto, Japan; 16 Department of Immunology, Graduate School of Medical Sciences, Faculty of Life Sciences, Kumamoto University 221481 , Kumamoto, Japan; 17 Division of Pathogenesis and Disease Regulation, Department of Pathology, Shiga University of Medical Science 13051, Otsu, Japan; 18 Global Station for Biosurfaces and Drug Discovery, Hokkaido University 12810, Sapporo, Japan; 19 Department of Vaccine Immunology, Hokkaido University Graduate School of Medicine 38251, Sapporo, Japan; St. Jude Children's Research Hospital, Memphis, Tennessee, USA

**Keywords:** ARNAX, TLR3, adjuvant, COVID-19

## Abstract

ARNAX is a synthetic nucleotide-based Toll-like receptor 3 (TLR3) ligand that specifically stimulates the TLR3/TIR domain-containing adaptor molecule 1 (TICAM-1) pathway without activating inflammatory responses. ARNAX activates cellular immunity via cross-presentation; hence, its practical application has been demonstrated in cancer immunotherapy. Given the importance of cellular immunity in virus infections, ARNAX is expected to be a more effective vaccine adjuvant for virus infections than alum, an adjuvant approved for human use that mainly enhances humoral immunity. In the present study, the trimeric recombinant spike protein of severe acute respiratory syndrome coronavirus 2 (SARS-CoV-2) was prepared as a vaccine antigen and formulated with ARNAX. When T-cell and neutralizing antibody responses were evaluated in immunized mice, antigen formulated with ARNAX generated significantly larger numbers of antigenspecific CD4^+^ and CD8^+^ T cells, as well as higher titers of neutralizing antibodies, compared to antigen alone or antigen formulated with alum. In experiments where immunized mice were challenged with a SARS-CoV-2 mouse-adapted virus derived from the ancestral strain, immunization with antigen formulated with ARNAX reduced virus titers in the lungs at 3 days post-infection to a much greater extent than did immunization with either antigen alone or that formulated with alum. These results show that ARNAX potently enhances the levels of both cellular and humoral immunity above those seen with alum, providing significantly greater viral clearing responses. Thus, ARNAX may act as a useful adjuvant for prophylactic vaccines, particularly for viral infectious diseases.

**IMPORTANCE:**

Cellular immunity is a critical immunological defense system against virus infections. However, aluminum salts, the most widely used adjuvant for vaccines for human use, do not promote strong cellular immunity. To prepare for the next pandemic of viral origin, the development of Th1-type adjuvants with low adverse reactions that induce cellular immunity is necessary. ARNAX is a TLR3 agonist consisting of DNA-RNA hybrid nucleic acid, which is expected to be an adjuvant that induces cellular immunity. The present study using a coronavirus disease 2019 mouse model demonstrated that ARNAX potently induces cellular immunity in addition to humoral immunity with minimal induction of inflammatory cytokines. Therefore, ARNAX has the potential to be used as a potent and welltolerated adjuvant for vaccines against pandemic viruses emerging in the future.

## INTRODUCTION

Vaccination is the most effective countermeasure for the control of infectious diseases, and an adjuvant is a key component in a variety of vaccines, especially subunit vaccines, to enhance protective antigen-specific immune responses. Since the induction of neutralizing antibodies is an essential component of the effectiveness of vaccines, the main focus of vaccine and adjuvant evaluation is on the ability to induce such antibodies. However, neutralizing antibodies can be less effective if antigenic variants arise, as continually observed with viral infections such as influenza. Since the 1920s, aluminum salts (alum) have been widely used as an adjuvant in human vaccines and remain the mainstay (1, 2). Although alum is safe and well tolerated in humans, the adjuvant activity is quite mild and tends to shift the Th1/Th2 balance of the resulting immune response toward Th2 polarization, resulting in suboptimal induction of cell-mediated immunity (3–6). Since cellular immunity is important to provide coverage against antigenic variants that arise as viruses circulate in the human population (7, 8), the development of Th1-type adjuvants that induce cellular immunity is required.

Dendritic cells (DCs) play a pivotal role for activation of acquired immunity through antigen presentation (9, 10). Recognition of pathogen-associated molecular patterns of an invading pathogen by pattern recognition receptors (PRRs) on or within DCs is essential for priming specific T cells (11–13). This recognition event promotes the efficient processing of antigens acquired by the DC from the pathogen and presentation of these antigen fragments on the DC surface in association with major histocompatibility complex (MHC) molecules. Also triggered is the upregulation of co-stimulatory molecules which, together with the antigen-MHC complexes, are recognized by receptors on naïve T cells, leading to their activation. The accompanying cytokine production by the DC influences the nature of the resulting activated T cells that are produced.

The Toll-like receptors (TLRs) are a major type of PRR (10, 14, 15), and thus, TLR agonists are of great interest in vaccine research for their ability to enhance acquired immunity, acting as potent adjuvants (15–18). In particular, some TLRs that recognize viral DNA or RNA are known to promote cross-presentation, which is the uptake of extracellular antigens by DCs, and induce antigen-specific cytotoxic T-lymphocyte (CTL) responses, a critical immune response especially against virus infection and cancer (19–21). Although TLR agonists have potent adjuvant activity, only a few have been licensed. This is likely because they may induce undesirable inflammation, leading to adverse events. One of the challenges in adjuvant development, therefore, is to modulate the inflammatory response while maintaining immune activity.

ARNAX is a Toll-like receptor 3 (TLR3) ligand that contains a double-strand RNA (dsRNA) structure capped at one 5′ end with a phosphorothioated GpC DNA, rather than CpG DNA (22, 23). The DNA structure at the 5′ end facilitates the transport of ARNAX to the endosome (24). The immune activation by ARNAX has been attributed to a dsRNA consisting of a defective interfering RNA sequence in the measles virus vaccine strain (25). Recognition of dsRNA occurs by both cytoplasmic and endosomal PRRs (26), with TLR3 being one such receptor primarily expressed on APCs such as DCs and macrophages (27–29). It recognizes ARNAX in endosomes, promotes the maturation of APCs, and activates innate immunity. In addition, TLR3 signaling induces cross-presentation in DCs and promotion of CTL responses (29). Taking advantage of this feature to induce Th1-type immunity, researchers have primarily developed ARNAX as an adjuvant for cancer immunotherapy (22, 23, 30–35). TLR3 activates the signaling pathway of Toll/interleukin-1 receptor (TIR) domain-containing adaptor molecule 1 (TICAM-1) (36), also named TIR domain-containing adaptor inducing interferon (IFN)-β (TRIF) (37), to induce type I IFNs. The TLR3-TICAM-1 signaling pathway does not induce the high level of pro-inflammatory cytokines as other TLR signaling pathways that involve the myeloid differentiation primary response 88 adaptor protein. In addition, the activation of the retinoic acid inducible gene-I (RIG-I)-like receptor (RLR) family of PRRs, such as melanoma differentiation-associated protein 5 (MDA5), RIG-I, and laboratory of genetics and physiology 2 (LGP2), by ARNAX stimulation is restricted in DCs because ARNAX is mainly transported to endosomes rather than to the cytoplasm, where these receptors reside. Activation of ubiquitously expressed RLRs by adjuvants such as poly I:C can induce systemic type I IFNs and pro-inflammatory cytokines and, as such, is of great concern in terms of adverse reactions (26). In this regard, the DNA/RNA hybrid adjuvant “ARNAX,” with its restricted signaling potential, is a unique TLR agonist.

Coronavirus disease 2019 (COVID-19), which is caused by severe acute respiratory syndrome coronavirus 2 (SARS-CoV-2), has repeated peaks of prevalence even after the World Health Organization declared the end of the “public health emergency of international concern.” Although mRNA vaccines have contributed to control of the pandemic, many concerns remain regarding their safety, such as the strong adverse reactions that are observed (38, 39). Therefore, alternatives to mRNA vaccines are required, particularly for repeated vaccination of children and the elderly. In this study, we evaluated the potency of ARNAX as an adjuvant to enhance the immunogenicity of a recombinant antigen of the SARS-CoV-2 spike (S) protein. ARNAX demonstrated a more significant enhancement of cellular and humoral immunity compared to alum in a mouse model. A safe and effective adjuvant, ARNAX, shows great potential not only for COVID-19 but also for future pandemics.

## RESULTS

### Cryo-electron microscopy confirms the recombinant spike protein antigen is trimeric

**Fig 1:**
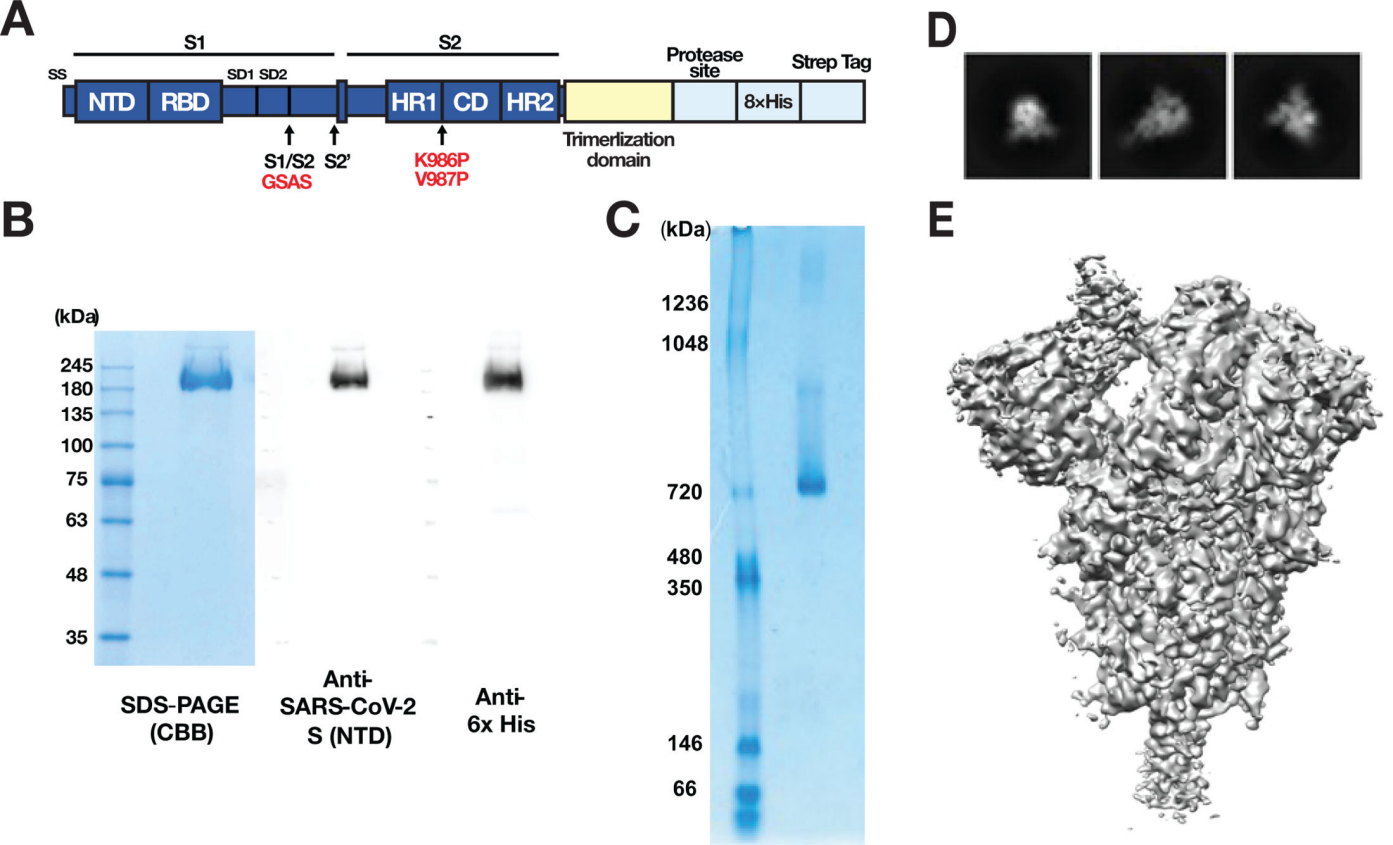
Preparation of recombinant SARS-CoV-2 trimeric spike protein as a vaccine antigen. (A) Schematic showing the construct used for the production of SARS-CoV-2 trimeric spike protein. (B) SDS-PAGE and Western blotting analysis of purified recombinant protein in reduced conditions. The standard of molecular weight is indicated at the left of the figure as kilodalton. The left picture shows a gel stained with Coomassie brilliant blue. Antibodies used for Western blotting were against SARS-CoV-2 spike S1 region (NTD) (the middle) and His-tag (the right). (C) Blue native-PAGE analysis of purified recombinant protein. The standard of molecular weight is indicated at the left of the figure as kilodalton. (D) Representative 2D classes obtained by CryoEM analysis. (E) The reconstructed 3D volume map from 31,432 particles.

A recombinant SARS-CoV-2 S protein was prepared using a CHO-DG44 protein expression system as the antigen to be formulated with the ARNAX adjuvant. The recombinant antigen is the ectodomain of the S protein (ancestral strain) with mutations for structural stability (2P mutations: K986P and V987P and substitution of the furin cleavage site RRAR to GSAS) and with a trimerization domain (T4 foldon) and Tag sequence at the C-terminus (Fig. 1A) (40). The purified antigen was analyzed by sodium dodecyl sulfate-polyacrylamide gel electrophoresis (SDS-PAGE) and Western blotting (Fig. 1B). A single band around 200 kDa was observed by SDS-PAGE and Coomassie blue staining. This size was similar to that reported previously (41). The protein band reacted with both an anti-SARS-CoV-2 S antibody against the N-terminal domain (NTD) of the S protein and an anti-His antibody, indicating that the protein retains almost its entire length from the N-terminus to the C-terminus, harboring the NTD of S protein and His tags, respectively. The protein was also analyzed by native PAGE (Fig. 1C). A major band was detected at approximately 720 kDa, implying the antigen is a trimer. To confirm the trimeric structure of the antigen, the protein was subjected to cryo-electron microscopy (cryo-EM) (Fig. 1D and E; Table S1). The molecular structure of the antigen was reconstructed at 3.49 Å resolution from two-dimensional (2D) classification images of 31,432 particles (three representative classes are shown in Fig. 1D). The reconstruction showed a trimetric structure with a one-up state of the receptor-binding domain (Fig. 1E). The S antigen (10 µg/dose) was formulated with ARNAX (10 µg/dose), alum (1 mg/dose), or no adjuvant. These preparations (hereafter referred to as S plus ARNAX, S plus alum, and S alone, respectively) were used to immunize C57BL/6 mice. S plus alum was delivered by the intramuscular route as is usual for vaccines containing this adjuvant, whereas S plus ARNAX and S alone were delivered subcutaneously as in previous studies (33, 35).

### ARNAX does not promote the excessive systemic cytokine and chemokine response observed with the TLR3 agonist poly I:C

**Fig 2:**
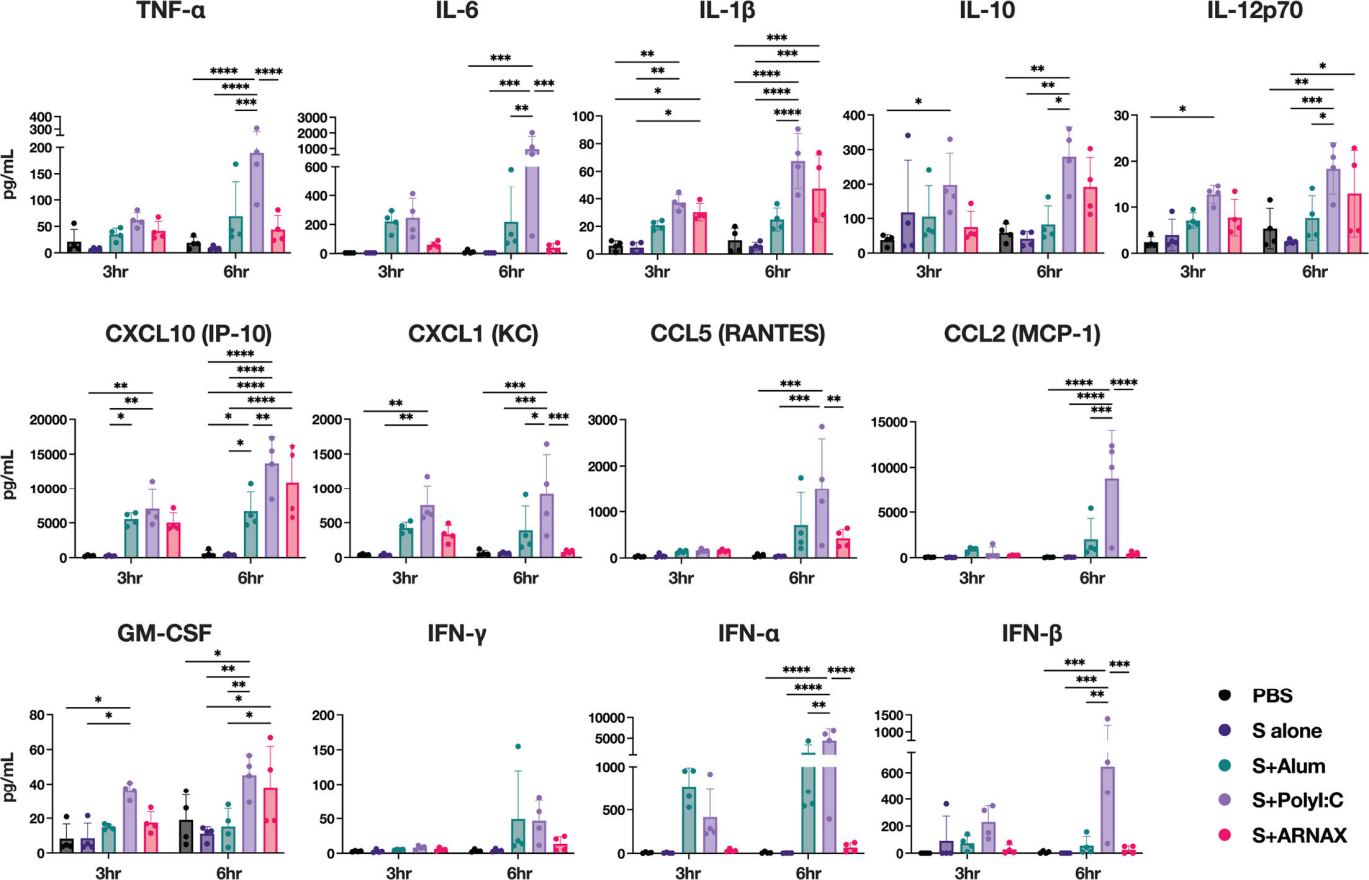
Cytokine and chemokine levels 3 or 6 h after vaccination. Sera were collected from immunized mice at 3 or 6 h after the first dose of vaccine, and concentrations of TNF-α, IL-6, IL-1β, IL-10, IL-12p70, IP-10, KC, RANTES, MCP-1, GM-CSF, IFN-γ, IFN-α, and IFN-β were measured using a bead-based immunoassay (*n* = 4). Individual samples are shown as dots; horizontal lines indicate the median. Significance was calculated using one-way analysis of variance with Tukey’s multiple comparison test. *P < 0.05, **P < 0.01, ***P < 0.001, ****P < 0.0001.

The systemic induction of cytokines and chemokines was examined in serum samples collected from mice 3 or 6 h after a single inoculation of the different antigen formulations. poly I:C, a dsRNA analog recognized by TLR3 and RLRs, was also inoculated into mice with S as a positive control (10 µg/dose, hereafter referred to as S plus poly I:C). Cytokines and chemokines measured were the following: tumor necrosis factor-α (TNF-α), interleukin (IL)-6, IL-1β, IL-10, IL-12p70, interferon (IFN)-γ-inducible protein 10 (IP-10, also called CXCL10), keratinocyte-derived chemokine (KC, also called CXCL1), regulated on activation normal T cell expressed and secreted (RANTES, also called CCL5), monocyte chemotactic protein 1 (MCP-1, also called CCL2), granulocyte-macrophage colony-stimulating factor (GM-CSF), IFN-γ, IFN-α, and IFN-β (Fig. 2). As expected, all cytokines and chemokines except IFN-γ were significantly increased at 6 h post-vaccination in the serum of mice inoculated with S plus poly I:C compared to S alone, with particularly high levels of TNF-α, IL-6, KC, RANTES, MCP-1, IFN-α, and IFN-β. In the serum of mice inoculated with S plus ARNAX at 6 h post-vaccination, the levels of IL-1β, IL-12p70, IP-10, and GM-CSF were significantly higher compared to S alone but slightly lower than S plus poly I:C. In addition, the levels of TNF-α, IL-6, MCP-1, and KC were significantly lower with S plus ARNAX than with S plus poly I:C. Notably, there was no significant difference between S formulated with ARNAX or alum, the adjuvant widely used for its safety profile. These results suggest that, unlike poly I:C, ARNAX does not induce an excessive inflammatory response despite being a TLR3 ligand.

### Antigen-specific T-cell responses in mice immunized with S plus ARNAX are superior to S plus alum

**Fig 3:**
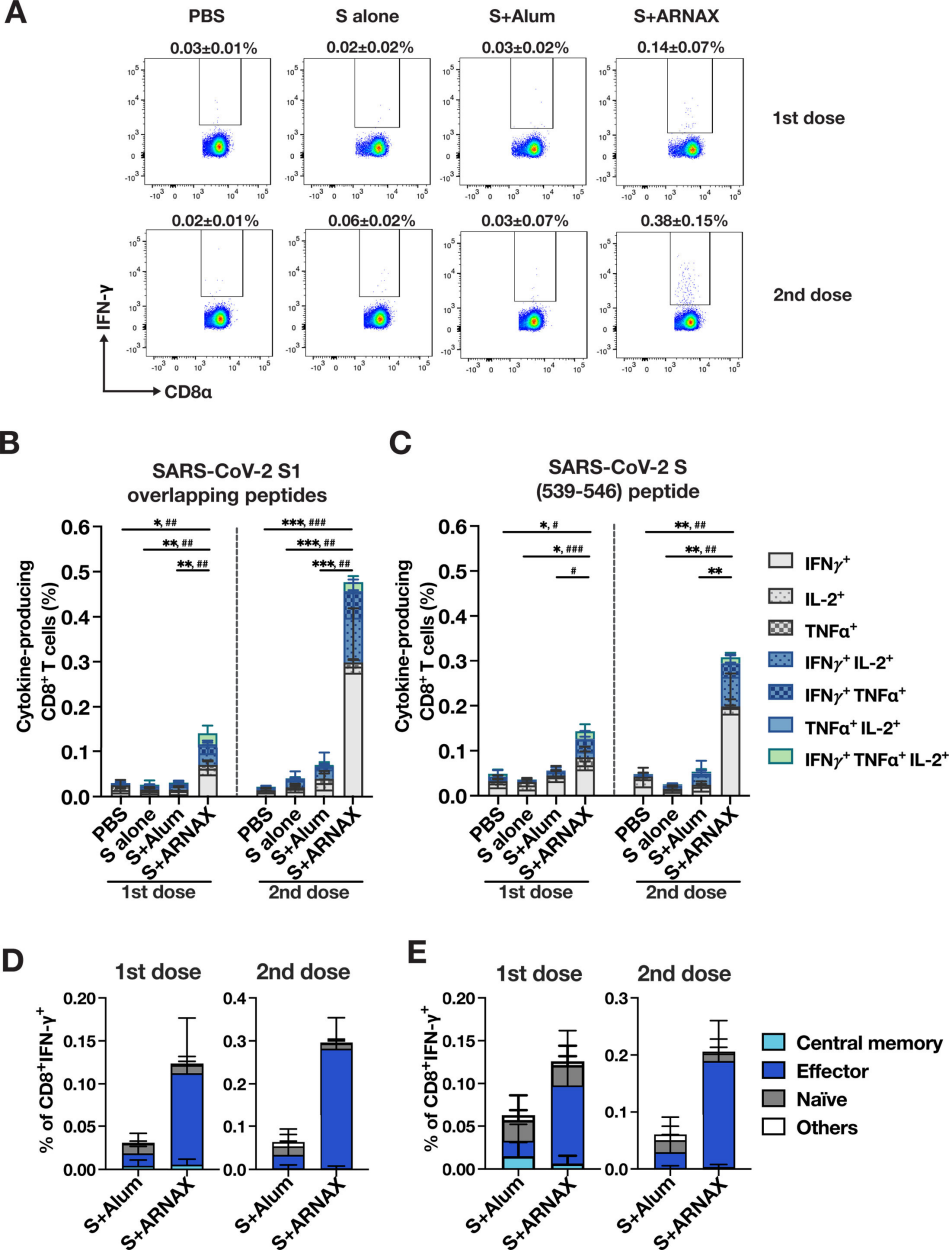
Analysis of antigen-specific CD8^+^ T-cell responses. Mice were immunized with each vaccine (*n* = 7–10). Ten days after the first dose or 4 days after the second dose of vaccine, splenocytes were harvested and re-stimulated with overlapping peptides for the SARS-CoV-2 spike S1 region. CD8^+^ T cells secreting IFN-γ, IL-2, and TNF-α were analyzed by intracellular cytokine staining using flow cytometry. (A) Representative results of IFN-γ^+^ CD8^+^ T cells are shown. The percentages of the IFN-γ-positive population in CD8^+^ T cells and the median ± SD for each group are shown above each plot. (B and C) The percentages of single, double, and triple cytokine-producing cells in CD8^+^ T cells stimulated with overlapping peptides (B) or AA 539–546 peptides of the S protein (C) are indicated separately and displayed as a stacked bar. Individual cytokine patterns are represented by the following: light gray for IFN-γ^+^, light gray with small dots for IL-2^+^, light gray with large dots for TNF-α^+^, light blue with small dot for IFN-γ^+^ IL-2^+^, light blue with large dot for IFN-γ^+^ TNF-α^+^, light blue for TNF-α^+^ IL-2^+^, and green for IFN-γ^+^ TNF-α^+^ IL-2^+^. Each error bar represents the mean ± SD. Significance is calculated for total cytokine-producing cells and multiple cytokine-producing cells (colored bars) using the Brown-Forsythe analysis of variance followed by Dunnett’s T3 test. For total cytokine-producing cells, *P < 0.05, **P < 0.01, ***P < 0.001; for multiple cytokine-producing cells, ^#^*P* < 0.05, ^##^*P* < 0.01, ^###^*P* < 0.001. (D and E) Subsets of IFN-γ^+^ CD8^+^ T cells stimulated with overlapping peptides (D) or AA 539–546 peptides of the S protein (E) are defined as follows: central memory for CD44^+^CD62L^+^, effector/memory for CD44^+^CD62L^−^, naïve for CD44^−^CD62L^+^, and others for CD44^−^CD62L^−^. Each error bar represents the mean ± SD. The results in panels B–D are the combined data from three independent experiments (*n* = 7–10).

To evaluate T-cell immunity induced by each antigen formulation, intracellular cytokine staining was conducted using splenocytes collected from immunized mice 10 days after the first dose or 4 days after a second dose. Splenocytes were stimulated *ex vivo* with an overlapping peptide pool containing a total of 166 peptides (15 mers overlapping with 11 amino acids) covering the S1 region of SARS-CoV-2. After stimulation with each peptide, IFN-γ-producing T cells were evaluated by fluorescence-activated cell sorting (FACS) analysis. The representative results of IFN-γ production in splenic CD8^+^ T cells stimulated with the overlapping peptide pool are shown in Fig. 3A. In mice immunized with a single dose of S plus ARNAX, 0.14% ± 0.07% (group median ± SD) of the CD8^+^ T cells produced IFN-γ, whereas only 0.03% ± 0.02% of the CD8^+^ T cells produced IFN-γ in mice immunized with S plus alum. The percentage of IFN-γ-producing CD8^+^ T cells in mice immunized with S alone was 0.02% ± 0.02%, similar to the percentage 0.03% ± 0.01% from mice injected with phosphate-buffered saline (PBS). After the second dose, the percentage of IFN-γ-producing CD8^+^ T cells further increased to 0.38% ± 0.15% for S plus ARNAX, with only minimal or no increase in the other groups.

Since T cells producing multiple cytokines are considered to be multi-functional, the percentage of CD8^+^ T cells producing IFN-γ, TNF-α, and/or IL-2 was calculated by further dividing IFN-γ-positive and IFN-γ-negative CD8^+^ T cells into TNF-α- and IL-2-producing populations. After *in vitro* stimulation with the overlapping peptide pool, spleen cells from mice inoculated with S plus ARNAX showed a significantly greater percentage of cytokine-producing CD8^+^ T cells compared to spleen cells from S plus alum-inoculated mice. Approximately four- and sixfold differences were observed after both first and second inoculations, respectively (Fig. 3B). Furthermore, S plus ARNAX induced significantly higher percentages of multiple cytokine-producing cells than did S plus alum or S alone (colored areas in Fig. 3B). These results suggest that ARNAX induces quantitatively and qualitatively superior CD8^+^ T-cell responses than alum. Antigen-specific CTL analysis was also performed using a single peptide of SARS-CoV-2 S (539–546: VNFNFNGL), which binds to H-2 K^b^ MHC class I of C57BL/6 mice. The percentage of cytokine-producing CD8^+^ T cells in mice immunized with S plus ARNAX was significantly higher than that in mice immunized with S alone or PBS after the first and second doses, and the level increased after the second dose, as was observed in the experiments with overlapping peptides (Fig. 3C). The percentage of multiple cytokine-producing cells was also markedly elevated after the first dose (Fig. 3C). The activation status of T cells was also examined using flow cytometry to divide the IFN-γ-producing CD8^+^ T cells into the effector/memory subset (CD44^hi^CD62L^low^), central memory subset (CD44^hi^CD62L^hi^), and naïve subset (CD44^low^CD62L^hi^). As expected from the time period after immunization, most of the IFN-γ-producing CD8^+^ T cells reactive to either the pool of peptides or the single peptide were effector/memory T cells in mice vaccinated with S plus ARNAX (Fig. 3D and E).

**Fig 4:**
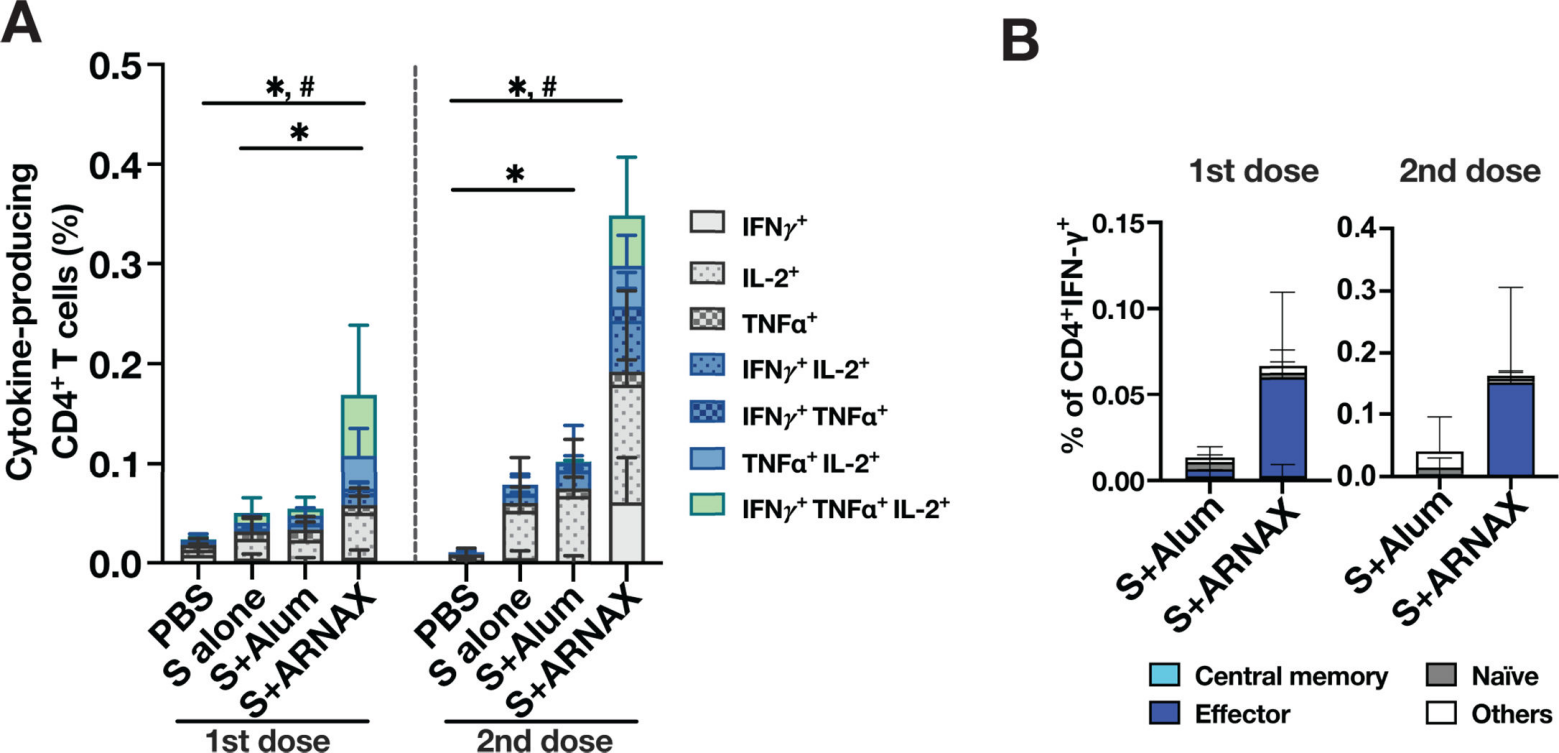
Analysis of antigen-specific CD4^+^ T-cell responses. CD4^+^ T cells secreting IFN-γ, IL-2, and TNF-α were analyzed as in panel B. CD4^+^ T cells producing IFN-γ, IL-2, and TNF-α were analyzed by intracellular cytokine staining using flow cytometry. (A) The percentages of single, double, and triple cytokines out of IFN-γ-, IL-2-, and TNF-α-producing cells in CD4^+^ T cells stimulated with overlapping peptides are indicated separately and displayed in a stacked bar. Each bar displays the following: light gray for IFN-γ^+^, light gray with small dots for IL-2^+^, light gray with large dots for TNF-α^+^, light blue with small dot for IFN-γ^+^ IL-2^+^, light blue with large dot for IFN-γ^+^ TNF-α^+^, light blue for TNF-α^+^ IL-2^+^, and green for IFN-γ^+^ TNF-α^+^ IL-2^+^. Error bars represent the mean ± SD. Significance is calculated for total cytokine-producing cells and multiple cytokine-producing cells (colored bars) using Brown-Forsythe analysis of variance followed by Dunnett’s T3 test. For total cytokine-producing cells, *P < 0.05; for multiple cytokine-producing cells, ^#^*P* < 0.05. (B) Subsets of IFN-γ^+^CD4^+^ T cells are defined as follows: central memory for CD44^+^CD62L^+^, effector/memory for CD44^+^CD62L^−^, naïve for CD44^−^CD62L^+^, and others for CD44^−^CD62L^−^. Error bars represent mean ± SD. These results are the combined data from three independent experiments (*n* = 7–10).

The cytokine (IFN-γ, TNF-α, and/or IL-2)-producing CD4^+^ T cells in spleens after stimulation with the overlapping peptide pool were also analyzed by intracellular cytokine staining (Fig. 4A). After the first and second immunizations, mice treated with S plus ARNAX induced a remarkably higher percentage of cytokine-producing CD4^+^ T cells than did S plus alum, with multiple cytokine-producing cells accounting for approximately 50% of the population. The tendency was similar to the results for cytokine-producing CD8^+^ T cells. The T-cell activation status of IFN-γ-producing CD4^+^ T cells was also examined by CD44 and CD62L staining (Fig. 4B). As observed for CD8^+^ T cells, the majority of IFN-γ-producing CD4^+^ T cells after inoculation of S plus ARNAX were effector/memory T cells.

In addition to the spleen, antigen-specific T-cell responses were similarly evaluated in the inguinal lymph nodes, which are closer to the site of vaccination than the spleen. Cells from all inguinal lymph nodes of three to four mice in each group were pooled as a single sample and subjected for intracellular cytokine staining. In contrast to the results for CD8^+^ T cells in the spleens, the percentages of cytokine-producing CD8^+^ T cells stimulated with either the peptide pool or the single peptide after the first dose were not significantly different among all vaccination groups (Fig. S1A and B). Although there was no noticeable induction of CTL in any group of mice in the lymph nodes after the first dose, the percentage of cytokine-producing CD8^+^ T cells in the S plus ARNAX immunization group was significantly higher than those in the other groups after the second dose (Fig. S1A and B). Cytokineproducing CD4^+^ T cells stimulated with the overlapping peptides in the lymph nodes were also examined (Fig. S1C). Consistent with the results of cytokine-producing CD8^+^ T cells, the mice vaccinated with S plus ARNAX showed a significantly stronger induction of cytokine-producing CD4^+^ T cells than the other groups after the second dose. After the first vaccination, a high percentage of antigen-specific CD8^+^ T cells were detected in the spleen but not in the lymph nodes. This may be attributed to differences in the primary sites of CD8^+^ T-cell accumulation at different times and under different conditions. Taken together with the results from splenocytes, the findings with lymph node antigen-specific T cells show that those induced by S plus ARNAX are quantitatively and functionally superior to those induced by S plus alum. This indicates that ARNAX is a more potent adjuvant for inducing cellular immunity than alum.

Also examined was the percentage of T follicular helper (Tfh) cells (defined as CD44^hi^, PD-1^+^, and CXCR5^+^ in CD4^+^ T cells) in lymph nodes as these provide critical help to B cells during the germinal center reaction to facilitate generation of protective humoral immunity. The percentage of Tfh cells in the lymph nodes increased after the second dose in the S plus ARNAX immunization group but not after immunization with S alone or S plus alum (Fig. S1D). This suggests that the adjuvant effect of ARNAX may also contribute to the induction of antibody titers.

### Humoral immunity induced by S plus ARNAX has a Th1 bias

**Fig 5:**
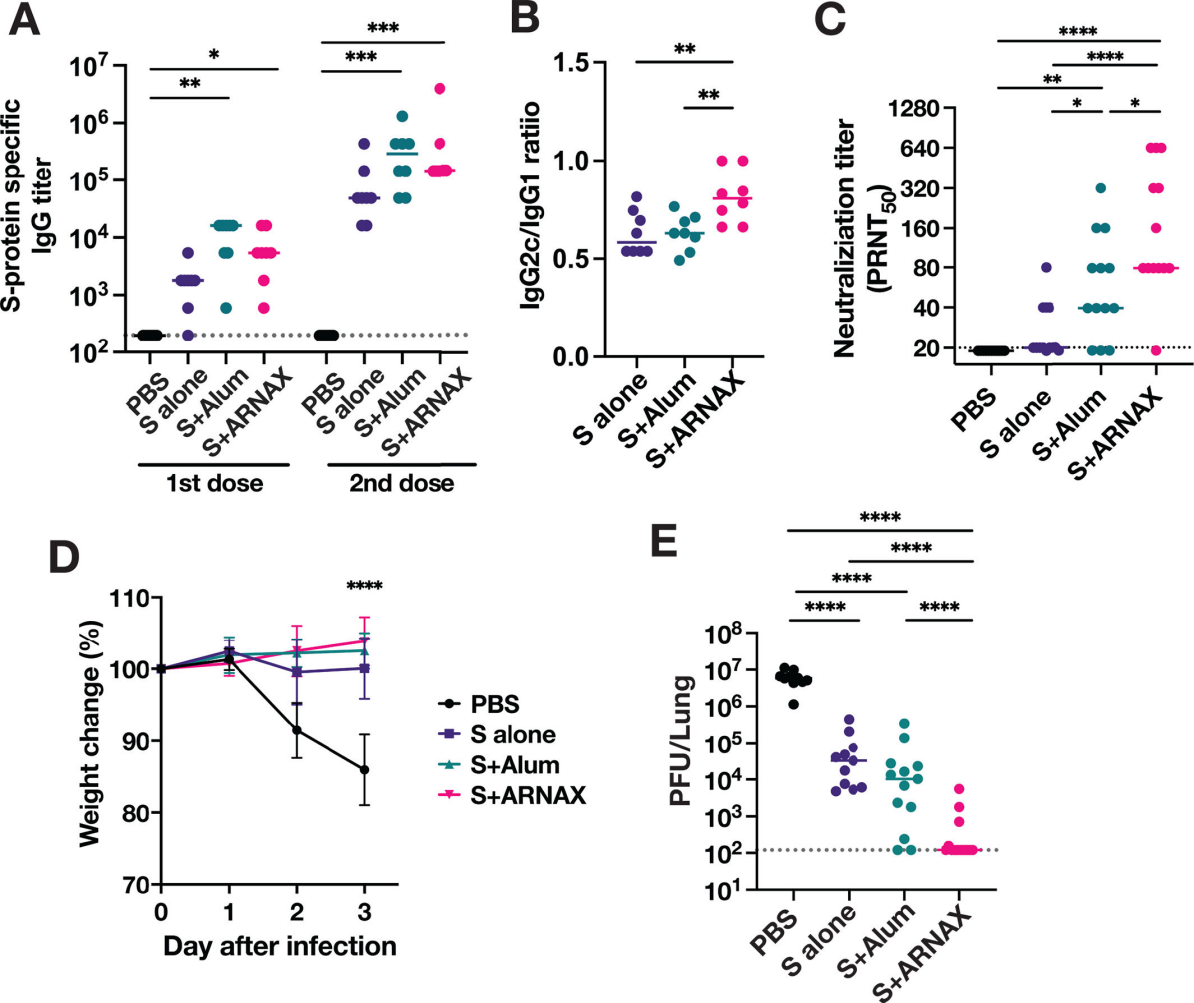
Antibody responses and protective effect of S plus ARNAX in mice against SARS-CoV-2 infection. Serum samples were collected 2 weeks after both the first dose and the second dose of the vaccines. (A) Total IgG antibodies against SARS-CoV-2 S antigen were evaluated by ELISA (*n* = 8). (B) IgG2c and IgG1 antibodies against SARS-CoV-2 S antigen were evaluated by ELISA from sera taken after the second dose (*n* = 8), and IgG2c/IgG1 ratios were calculated. (C) Neutralizing antibody titers were evaluated from serum sampled at 2 weeks after the second dose by plaque reduction neutralization assay (*n* = 13). Dots represent individual mice; the accompanying horizontal lines indicate the median. Significance of the difference between groups, displayed above the columns, is calculated using one-way analysis of variance (ANOVA) with Tukey’s multiple comparison test. *P < 0.05, **P < 0.01, ***P < 0.001, ****P < 0.0001. Mice were challenged with 1 × 10^5^ PFU of SARS-CoV-2 mouse-adapted strain 2 weeks after the second dose. (D) The body weight of the mice was monitored daily after virus challenge and reported as a percentage of the starting weight (*n* = 8). All significant differences between the PBS control group and each vaccination group at 3 days post-infection (dpi) were ****P < 0.0001. (E) The virus titers in the lungs at 3 dpi were determined by plaque assay (*n* = 13). Dots represent individual mice; the accompanying horizontal lines indicate the median. Significance of the difference between groups, displayed above the columns, was calculated using one-way ANOVA with Tukey’s multiple comparison test. *P < 0.05, **P < 0.01, ***P < 0.001, ****P < 0.0001.

Next, antibody titers were examined in mice immunized twice with S plus ARNAX, S plus alum, S alone, or PBS. Sera were collected 2 weeks after each vaccination, and antigen-specific IgGs were evaluated by enzyme-linked immunosorbent assay (ELISA). Antigen-specific total IgG was detected after the first dose, with a median of total IgG titers of 5,400 for S plus ARNAX and 16,200 for S plus alum, both higher than 1,800 for S alone (Fig. 5A). After the second dose, antigen-specific total IgG titers in all mice except mice inoculated with PBS increased to more than 20,000. The IgG titers in mice vaccinated with S plus ARNAX and S plus alum remained significantly higher than in the S alone group: median IgG titers of 145,800 in the S plus ARNAX group; 291,600 in the S plus alum group; and 48,600 in the S alone group (Fig. 5A). These results confirm the adjuvant effect of ARNAX on humoral immunity.

Since Th1 and Th2 polarity in mice can be roughly evaluated by the ratio of IgG2c to IgG1, antigen-specific IgG2c and IgG1 were examined by ELISA using sera collected after the second vaccination, and IgG2c:IgG1 ratios were calculated (Fig. 5B). As expected, the IgG2c/IgG1 values in the S plus ARNAX group were significantly higher than those in the S plus alum group, suggesting that ARNAX more preferably induced Th1 polarization compared to alum.

We also conducted a plaque reduction neutralization test (PRNT) to evaluate neutralizing antibody titers in these sera. Neutralizing antibodies were not detected in any of the groups after one dose. After two doses of S plus ARNAX, neutralizing antibodies were above 80 in all but one mouse, with a median of 80, whereas those in mice immunized with S plus alum were below 80 in more than half of the mice, with a median of only 40 (Fig. 5C). Notably, neutralizing antibody titers were significantly higher in the S plus ARNAX group than in the S plus alum group, even though specific IgG titers were comparable. These results suggest that ARNAX can induce not only cellular immunity but also humoral immunity. The discrepancy between antigen-specific IgG and neutralizing antibody titers may have resulted from the induction of affinity maturation of B cells in the germinal center by TLR3 signaling (42) or differences in the form of the antigen in the different formulations. These are consistent with the superiority of Tfh cell levels induced in the ARNAX group (Fig. S1D). Collectively, given the effect on the induction of cellular immunity, our findings suggest that ARNAX is a potent and well-balanced adjuvant that can simultaneously induce cellular and liquid immunity.

### Protective effect against SARS-CoV-2 infection in mice immunized with S plus ARNAX

To investigate the protective effect of adjuvants formulated with S antigen, mice were inoculated intranasally with 1 × 10^5^ PFU of SARS-CoV-2 mouse-adapted strain (SARS-CoV-2 MA-P10) 2 weeks after the second vaccination. Weight changes were monitored daily until 3 days post-infection (dpi) (Fig. 5D), and virus titers in the lungs at 3 dpi were determined by plaque assay (Fig. 5E). After infection, mice in the non-vaccinated (PBS) group lost more than 10% of their body weight, while vaccinated groups showed no weight loss. Non-vaccinated mice had viral titers of approximately 10^7^ PFU at 3 dpi, while the virus titers in mice vaccinated with S plus ARNAX were less than 10^3^ PFU, with the values of five out of eight mice being below the detection limit. The virus titers in the S plus ARNAX group were significantly lower than those in any other group. This potent suppressive effect on viral replication in the lungs induced by immunity to S plus ARNAX was compatible with the results of combined CTL and neutralizing antibody responses (Fig. 3 and 5C).

Since it has been reported that cytokine and chemokine levels in serum are closely related to the severity of the disease (43), cytokines and chemokines were measured in the sera at 3 days after infection in each vaccination group (Fig. S2). Concentrations of IP-10 and KC were significantly higher in the PBS control group than in the other groups, presumably due to responses to viral replication in the lungs. Despite no increase in IL-1β in the PBS group, the alum group had significantly higher IL-1β levels than the PBS and ARNAX group. TNF-α, IL-10, RANTES, and IFN-β also showed similar trends. These findings suggest that immunization with the ARNAX adjuvant results in a greater diminution of inflammation after subsequent infection than immunization with the alum adjuvant.

### Protection against heterologous SARS-CoV-2 infection in mice immunized with XBB1.5 variant S antigen and ARNAX

**Fig 6:**
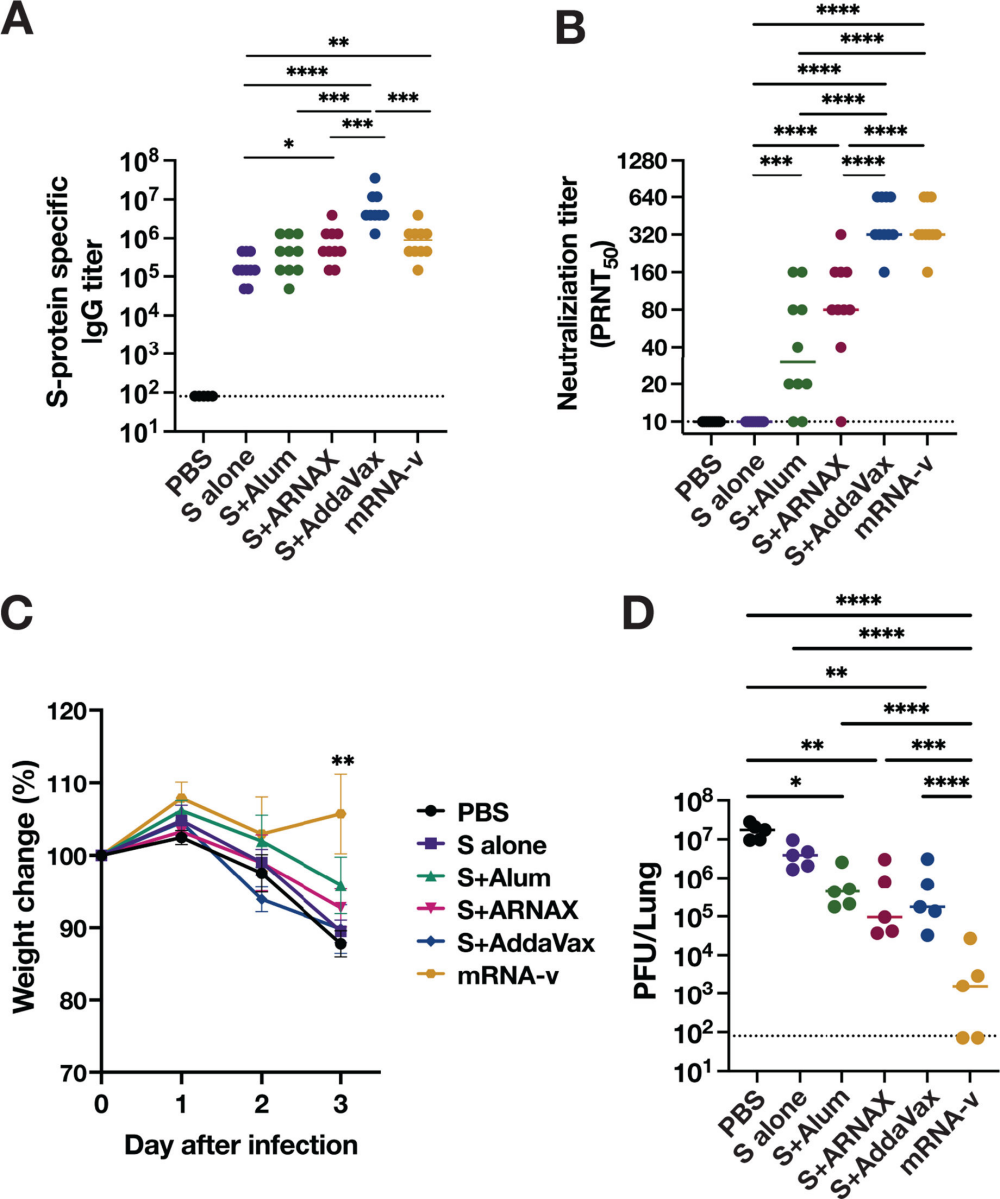
Protective effect of XBB1.5 S antigen plus ARNAX in mice against heterologous SARS-CoV-2 infection. Serum samples were collected 2 weeks after the second dose of the vaccines. (A) Total IgG antibodies against SARS-CoV-2 S antigen were evaluated by ELISA (*n* = 10). (B) Neutralizing antibody titers were evaluated by plaque reduction neutralization assay (*n* = 10). Dots represent individual mice; the accompanying horizontal lines indicate the median. Significance of the difference between groups, displayed above the columns, is calculated using one-way ANOVA with Tukey’s multiple comparison test. *P < 0.05, **P < 0.01, ***P < 0.001, ****P < 0.0001. Mice were challenged with 1 × 10^5^ PFU of SARS-CoV-2 mouse-adapted strain 2 weeks after the second dose. (C) The body weight of the mice was monitored daily after virus challenge and reported as a percentage of the starting weight (*n* = 5). All significant differences between the mRNA vaccine group and the other groups at 3 dpi were **P < 0.01. (D) The virus titers in the lungs at 3 dpi were determined by plaque assay (*n* = 5). Dots represent individual mice; the accompanying horizontal lines indicate the median. Significance of the difference between groups, displayed above the columns, was calculated using one-way ANOVA with Tukey’s multiple comparison test. *P < 0.05, **P < 0.01, ***P < 0.001, ****P < 0.0001.

To determine the cross-protective effect of each vaccination, mice were vaccinated twice with the S antigen of the SARS-CoV-2 XBB1.5 variant and challenged with the heterologous MA-P10 strain derived from the ancestral virus. The S antigen was formulated with alum, ARNAX, and also AddaVax, an MF59-like adjuvant which is used in some current vaccines for respiratory viruses. Although antigen amounts cannot be matched to the other groups, the currently available mRNA vaccine for the XBB variant was also included as an additional comparison in this experiment. The antibody titers in the serum were examined 2 weeks after the second dose of vaccine (Fig. 6A). Antigen-specific IgGs in mice vaccinated with S plus AddaVax were significantly higher than those in the other groups. Antigen-specific IgGs in mice vaccinated with S plus ARNAX were comparable to those in mice vaccinated with the mRNA vaccine. Neutralizing antibody titers against the XBB1.5 variant in the S plus AddaVax and mRNA vaccine groups (medians of 320) were significantly higher than those in the ARNAX group (a median of 80) (Fig. 6B). On the other hand, neutralizing antibody titers against the challenge strain, SARS-CoV-2 MA-P10, were not detected in any of the vaccination groups. Three days after infection with SARS-CoV-2 MA-P10, weight loss was observed in all vaccination groups at 3 dpi except for the mRNA vaccination group (Fig. 6C). Consistent with these results, virus titers in the lungs of mRNA-vaccinated mice on day 3 after heterologous challenge were the lowest of all groups, dropping to approximately 10^3^ PFU (Fig. 6D). Virus titers in the lungs were comparable between the AddaVax and ARNAX groups at approximately 10^5^ PFU, 100 times lower than in the PBS (unvaccinated) group (Fig. 6D). These results may imply that factors other than antibodies contribute to the decrease in virus titers in the lungs in the ARNAX and mRNA vaccine groups.

To further investigate whether the viral suppression of mRNA vaccines in the lungs is due to cellular immunity, intracellular cytokine staining was conducted using splenocytes and LN cells collected from immunized mice 4 days after a second dose. The percentage of cytokine-producing CD8^+^ T cells in the S plus ARNAX group was approximately 0.6%, compared to 2.5% in the mRNA vaccine group, a fourfold higher percentage (Fig. S3A, left panel). This strong induction of CTLs by the mRNA vaccine may be the reason for the low titer of the virus in the lungs. On the other hand, the percentage of cytokine-producing CD4^+^ T cells in the S plus ARNAX group was approximately 0.5%, whereas the percentage in the vaccine group was 0.25%, half of that of the S plus ARNAX group (Fig. S3A, right panel). In LNs, the percentage of cytokine-producing CD8^+^ T cells in the S plus ARNAX group was approximately 0.25%, compared to 0.3% in the mRNA vaccine group, a relatively similar percentage (Fig. S3B, left panel). The percentage of cytokine-producing CD4^+^ T cells in the S plus ARNAX group was approximately 0.5%, whereas the percentage in the mRNA vaccine group was 0.1%, one-fifth of that of the S plus ARNAX group (Fig. S3B, right panel). In addition, the percentage of Tfh in CD4^+^ T cells and germinal center B (GC B, defined as GL7^+^, Fas^+^, and B220^+^) cells in CD19^+^ CD45^+^ B cells in the LNs was examined. The percentage of Tfh and GC B in the S plus ARNAX group was about twofold higher than that in the mRNA vaccine group (Fig. S3C), which is consistent with the results in cytokine-producing CD4^+^ T cells (Fig. S3A and B). These results suggest that the ARNAX vaccine induces a greater number of Tfh and GC B cells in LNs, which are important for immune memory, than does the mRNA vaccine.

To assess the inflammatory status of the lungs after infection, the histopathology of lungs was examined by hematoxylin-eosin (H&E) staining using lung sections collected at 3 dpi (Fig. S4). Increased infiltration of immune cells in the peribronchiolar region was observed in the mice of the vaccinated groups. Immune cell infiltration was quite pronounced in the S plus AddaVax group. In contrast, these signs of inflammation were hardly observed in mice injected with PBS. These results suggest that the lung pathology at an early stage after infection in vaccinated mice is caused by infiltration of immune cells such as lymphocytes primed by the vaccine into infected areas of the lungs, particularly in the bronchioles. Thickening of the alveolar septa was clearly observed in mice in PBS, as well as the S alone and S plus alum groups. Some mice in the S plus AddaVax and mRNA vaccine groups showed severe inflammation in parts of the lungs. In addition, characteristic peri-bronchial edema was observed in the mRNA vaccine group. These may be attributed to a greater propensity of AddaVax and mRNA vaccines to induce cytokines associated with inflammation.

**Fig 7:**
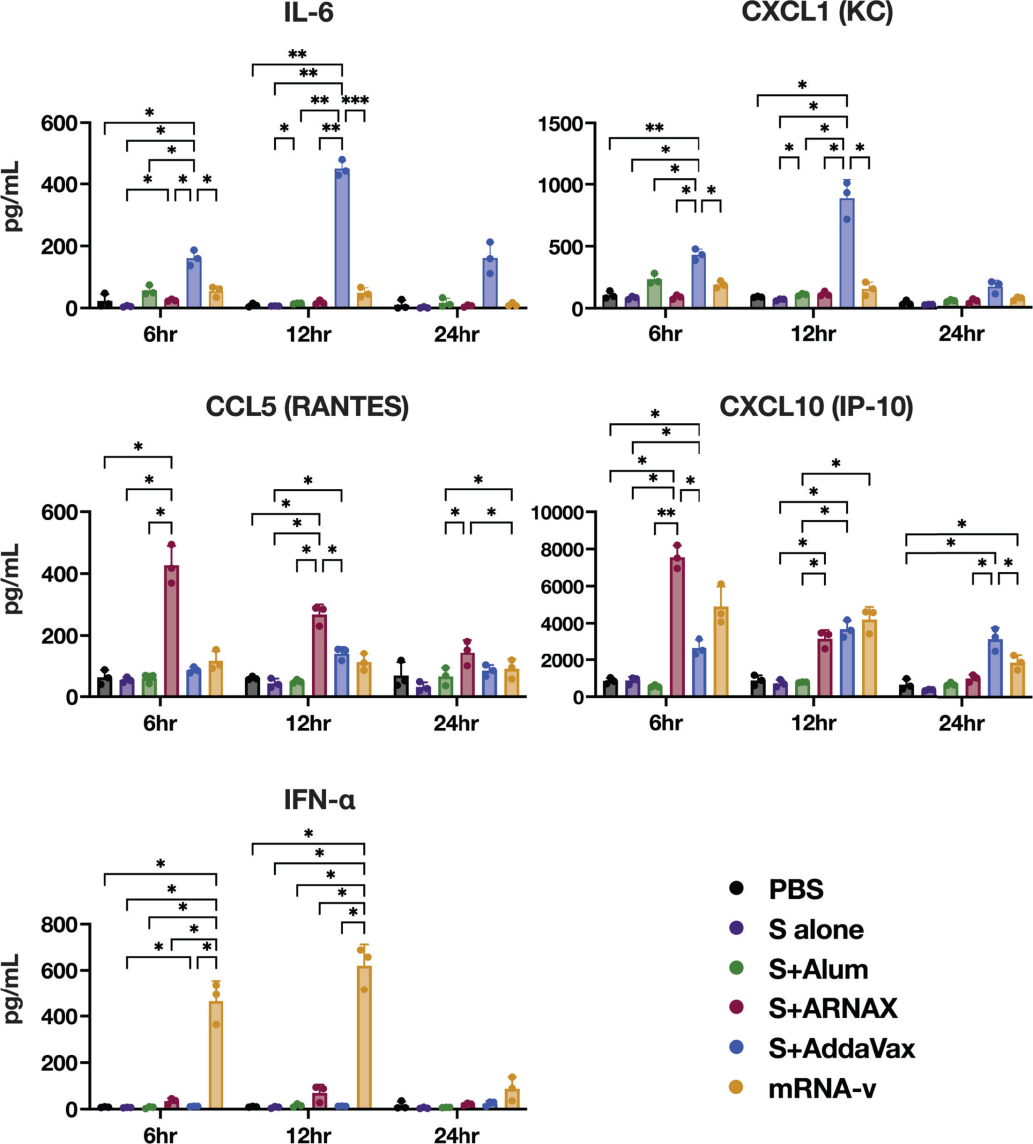
Cytokine and chemokine levels 6–24 h after vaccination. Sera were collected from immunized mice at 6, 12, and 24 h after the first dose of vaccine, and concentrations of the indicated cytokines and chemokines were measured using a bead-based immunoassay (*n* = 3). Individual samples are shown as dots; horizontal lines indicate the median. Significance was calculated using one-way ANOVA with Tukey’s multiple comparison test. *P < 0.05, **P < 0.01, ***P < 0.001.

Despite the potent immunogenicity of the mRNA and MF59 adjuvant vaccines, they have been reported to induce relatively strong febrile and local inflammation at the injection site (44–46). We therefore investigated whether these adverse reactions could be attributed to the profile of cytokines and chemokines induced 6–24 h after vaccination (Fig. 7; Fig. S5). The serum levels of the inflammatory cytokine IL-6 and chemokine KC were significantly higher in the AddaVax group compared to the other groups at 6 and 12 h after vaccination, and IFN-α was significantly higher in the mRNA vaccine group. RANTES and IP-10, chemokines involved in immune cell migration, were induced at the highest level in the ARNAX group. These data indicate that the pattern of cytokine chemokine induction 6–12 h after vaccination differs considerably among the ARNAX, AddaVax, and mRNA vaccine groups.

## DISCUSSION

The present study demonstrated the superiority of ARNAX over alum in terms of the induction of T-cell responses as well as neutralizing antibodies. This advantage of ARNAX could be due to the mechanism of activation of APCs to promote their antigen presentation. TLR agonists are known to exhibit potent adjuvant activity through direct activation of APCs, whereas alum does not. Although the mechanism of mild adjuvant activity with alum has not been fully elucidated, an antigen depot effect, whereby antigens are stored and slowly released from immunization sites, thereby facilitating antigen uptake by APCs, has been proposed as one of the mechanisms (47). In addition, it has recently been reported that alum triggers the release of danger-associated molecular patterns from cells around the injection site and induces local NLRP3-dependent inflammasome activation, resulting in prolonged inflammation and an intense polarization of Th1/Th2 balance toward Th2 immunity (3–6). On the other hand, TLR3 signaling polarizes the Th1/Th2 balance toward Th1 immunity. Indeed, S plus ARNAX and S plus poly I:C induced remarkable IP-10 (Fig. 2), which is particularly involved in the induction of Th1 immunity (48). The difference in Th1/Th2 polarization by S plus ARNAX and S plus alum may be attributed to these differences in the induction mechanism of innate immunity.

The induction of Th1 polarization by ARNAX was demonstrated by the pattern of IgG2c versus IgG1 antibody induction (Fig. 5B) and the induction of antigen-specific IFN-γ-producing CD4^+^ T cells (defined as Th1) by ARNAX, which was qualitatively and quantitatively superior compared to alum (Fig. 4A). Th1 polarization influences the induction of CTLs. Induction of CTLs by vaccines requires cross-presentation by APCs. Among several DC subsets, the conventional DC (cDC) subset expressing X-C motif chemokine receptor 1 (XCR1) is strongly involved in CD8^+^ T-cell activation via antigen cross-presentation (49–51). This subset corresponds to the CD8α^+^ or CD103^+^ cDC subset in the mouse (52) and is CD141^+^ cDC in humans (28). Notably, this XCR1-expressing DC subset predominantly expresses TLR3 (28, 52), which recognizes dsRNA such as ARNAX and primes CD8^+^ T cells via cross-antigen presentation (22). This suggests that ARNAX efficiently stimulates XCR1-expressing DCs specialized for cross-presentation via TLR3, leading to superior induction of antigen-specific CTLs. Indeed, there was a marked difference between the influences of ARNAX and alum on CTL responses in this study (Fig. 3). Cellular immunity such as CTLs is a critical defense system against intracellular pathogens such as viruses. To elicit potent anti-viral CTL responses, vaccines need to engage the cross-presentation pathway, and this requirement has been a hurdle in the development of vaccines for viruses to induce effective T-cell immunity. As described in the introduction section, ARNAX is designed to overcome this hurdle. ARNAX, possessing the DNA structure at the 5′ end, is transported into endosomes, which enables ARNAX to stimulate TLR3 localized in that compartment. Compared to antibody responses, CTL responses are expected to contribute to immunity across different viral variants. In fact, the amino acid sequence of the SARS-CoV-2 S peptide (539–546: VNFNFNGL) used to detect the CTL response in the study has been reported to be conserved among other coronaviruses (53, 54). Therefore, superior CD8^+^ T-cell responses reactive with the peptide observed in this study suggest a possibility that formulation of S antigen with ARNAX could provide valuable cross-immunity against a broad spectrum of strains. In virus infections, such as COVID-19 and influenza, in which antigenic variants continuously appear, adjuvants that can elicit CTLs have a great advantage. In addition to CTL, ARNAX exhibited a superior outcome to alum in the induction of neutralizing antibodies (Fig. 5C). It was reported that the germinal center formation is facilitated through the TLR3-TICAM-1 signal pathway (42). Affinity maturation of B cells in the germinal center promotes the generation of more potent neutralizing antibodies (55), which may contribute to the advantage of ARNAX in the induction of neutralizing antibodies. Thus, ARNAX is a more promising vaccine adjuvant for virus infections than alum.

Since TLR3 recognizes dsRNA such as poly I:C and its derivatives, as well as ARNAX, poly I:C and its derivatives can also induce CTL responses via the cross-presentation pathway. Therefore, the application of poly I:C and its derivatives in cancer immunotherapy and viral vaccines has been attempted in syngeneic mouse tumor transplant models and virus challenge mouse models (56–59). Despite the favorable results in animal models, clinical trials of poly I:C and its derivatives, especially the stabilized form (poly-ICLC), have made little progress due to severe side effects. In clinical trials, fever, hypotension, and arthralgia-myalgia syndrome were the most common side effects, followed by decreased blood counts, flu-like symptoms, renal failure, bone pain, and toxicity to the liver, bone marrow, and central nervous system (60–63). This is probably because poly I:C additionally activates the cytoplasmic dsRNA sensor, MDA5 (64, 65), resulting in systemic and robust production of type-I IFNs/cytokines that cause unwanted inflammation. In contrast to poly I:C, ARNAX induces limited inflammatory cytokine/IFN-β production in a TLR3-dependent manner rather than through the MDA5 pathway. Although the mechanism remains unclear, it is hypothesized that the GpC cap directs dsRNA to TLR3-positive DCs for endocytosis (22, 30, 66). These are consistent with the results in Fig. 2, suggesting that ARNAX is a safer adjuvant for infectious disease than poly I:C.

This study showed that the mRNA vaccine induced potent Th1-polarized immune responses. This is reasonable, given that mRNA vaccines can be delivered directly to the cytoplasm of APCs. Here, the encoded S proteins are translated, some of which can be processed and loaded onto MHC class I for antigen presentation to CD8^+^ T cells. As a result, mRNA vaccines strongly induce antigen-specific CD8^+^ T-cell responses (Fig. S3A and B). This CTL induction process differs from the ARNAX vaccine, which requires cross-presentation. Conversely, antigen presentation to CD4^+^ T cells requires uptake of extracellular antigens into endosomes of APCs and loading of degraded peptides onto MHC class II. This may contribute to the fact that the percentage of antigen-specific CD4^+^ T cells was higher in the S antigen plus ARNAX vaccine group than in the mRNA vaccine group (Fig. S3A and B). Probably due to differences in the process of antigen presentation between the ARNAX adjuvant and the mRNA vaccine, the percentage of Tfh and GC B in LNs in the S plus ARNAX group was higher than that in the mRNA vaccine group (Fig. S3C). This overly strong Th1-polarized immune response may be the reason for the relatively short-lasting neutralizing antibodies observed with mRNA vaccination (67, 68). Further research is needed to clarify the details of this issue.

While mRNA and MF59 adjuvant vaccines have potent immunogenicity, they have been reported to induce relatively strong febrile and local inflammation at the injection site (44–46). Although activation of innate immunity is essential for the development of an effective adaptive immune response, this can sometimes cause undesired side effects, such as febrile reactions, if certain cytokines are overproduced. In fact, inflammatory cytokines such as IL-1, IL-6, and TNF-α are involved in the exothermic reaction. IL-6, shown here to be produced at the highest levels by vaccines containing AddaVax, is especially known to play an important role in fever (69–71). In addition, IFN-α, produced at high levels by the mRNA vaccine, and IFN-β have also been reported to cause fever because the type I interferons stimulate thermoregulatory neurons via induction of opioid receptor agonists and/or prostaglandins in the brain (72–75). These facts and the experimental results in the present study (Fig. 7) may indicate, in part, the causes of the adverse reactions to MF59 adjuvant vaccines and mRNA vaccines observed in humans.

Despite the adverse reactions associated with mRNA vaccines, these remain the mainstay for prevention of COVID-19. Indeed, mRNA vaccines are useful in emergency situations, such as at the beginning of pandemics, because they can be manufactured as soon as the gene sequence is identified and have proven potency in the induction of neutralizing antibodies. However, the adverse reactions caused by mRNA vaccines seem to be serious and potentially harmful. As the SARS-CoV-2 virus continues to circulate within the global population, it may be time to consider alternatives. With new vaccine adjuvants and delivery modes continually being developed, there is the potential for vaccines to be available for selection according to the circumstances and preferences of the recipients. Therefore, consideration should be given to the balance between humoral immunity, cellular immunity, and adverse reactions to COVID-19 vaccines. ARNAX is a potential candidate for a vaccine adjuvant that meets immunogenicity and safety requirements. Since adjuvants can be produced in advance, the practical application of the ARNAX adjuvant would help us prepare for the next pandemic.

## MATERIALS AND METHODS

### Cells and viruses

Chinese hamster ovary (CHO)-DG44 cells (Thermo Fisher Scientific, Waltham, MA, USA) were used for antigen preparation of the SARS-CoV-2 S protein. The CHO-DG44 cells require hypoxanthine and thymidine (HT) to grow due to a lack of dihydrofolate reductase genes involved in nucleotide synthesis. The CD DG44 medium (Thermo Fisher Scientific), including HT that is optimized for dihydrofolate reductase-deficient cells, was used to maintain CHO-DG44 cells, and the CD OptiCHO medium (Thermo Fisher Scientific) without HT was used to select stably transfected cells with HT-expressing plasmids according to the manufacturer’s instructions. The transmembrane serine protease 2 (TMPRSS-2)-expressing Vero E6 (VeroE6/TMPRSS2) cells or TMPRSS- and angiotensin-converting enzyme 2-expressing Vero E6 (VeroE6/TMPRSS2-ACE2) cells were maintained in Dulbecco’s modified Eagle’s medium (DMEM) with low glucose (Thermo Fisher Scientific) supplemented with 10% heat-inactivated fetal calf serum (FCS) (Cytiva, Buckinghamshire, UK), 100 U/mL of penicillin, and 100 µg/mL of streptomycin (Sigma-Aldrich, St. Louis, MO, USA) at 37°C in 5% CO_2_. VeroE6/TMPRSS2 cells were used for the measurement of neutralizing antibody titers and virus titers.

SARS-CoV-2 WK-521 (Ancestral strain) and 1923–018 (XBB1.5.19) and the mouse-adapted SARS-CoV-2 MA-P10 were propagated in VeroE6/TMPRSS2 cells. To prepare stocks of SARS-CoV-2, the culture medium was centrifuged to separate the virus from cells and then stored at –80°C until use.

### Preparation of recombinant SARS-CoV-2 spike protein

The gene of semi-stabilized SARS-CoV-2 S protein with codon optimization for CHO cells was synthesized by GenScript (Piscataway, NJ, USA). The sequence of S protein was derived from an ancestral strain and modified according to previous reports (40, 41). The modification at the furin cleavage site from RRAR to GSAS and the K986P and V987P mutations in the fusion peptide were inserted for structural stabilization. The transmembrane domain and cytoplasmic tail were replaced with a T4 trimerization motif, followed by the addition of an HRV 3C protease site, a Hexa-His tag, and a Strep-tag II at the C-terminus. The construct for the SARS-CoV-2 S trimer (referred to as S antigen) was inserted into the pDC62c5-U53 plasmid, which is a vector optimized for a selection of transfectants by HT expression and protein expression in the CHO-DG44 cell systems.

The plasmid for expression of the S trimer was transfected into CHO-DG44 cells using FreeStyle MAX Reagent (Thermo Fisher Scientific). After 2 days of incubation at 37°C in 8% CO_2_ with shaking at 125 rpm, the medium was replaced with CD OptiCHO medium containing 4 mM GlutaMAX-I (Thermo Fisher Scientific) to select stable producer clones. The selected clone was then cultured in Dynamis AGT medium (Thermo Fisher Scientific) containing 8 mM GlutaMAX-I at 37°C in 8% CO_2_ with shaking at 125 rpm for 5 days to produce protein. The supernatant was centrifuged to remove cell debris, filtrated through a 0.2 µm pore filter (Thermo Fisher Scientific), and S antigen was purified using High-Capacity Ni-IMAC Resin (Thermo Fisher Scientific) according to the manufacturer’s instructions. The buffer solution of the eluted sample was replaced with PBS using a 100 K concentrator PES (Thermo Fisher Scientific). Protein concentration was determined with the Rapid Gold BCA Protein Assay Kit (Thermo Fisher Scientific). Purified S antigen was stored at −80°C until use.

### SDS-PAGE and Western blotting

Purified S antigen was mixed with NuPAGE LDS Sample Buffer (4×) (Thermo Fisher Scientific) at 3:1 in the presence of β-mercaptoethanol and heated at 70°C for 5 minutes. The proteins in the sample were separated by electrophoresis in 8% polyacrylamide gels, followed by staining with Coomassie brilliant blue or transferring onto 0.2 µm polyvinylidene difluoride (PVDF) membranes (Merck, Darmstadt, Germany) for Western blotting. For Western blotting, the membranes were soaked for 1 h at room temperature with 5% skim milk in tris-buffered saline containing 0.05% Tween 20 (TBS-T) to block non-specific antibody reactions. After washing three times with TBS-T for 5 minutes, the membranes were reacted with anti-His-Tag monoclonal antibody (TaKaRa Bio, Shiga, Japan) at a dilution of 1:2,000 or anti-SARS-CoV-2 spike antibody (HL6; GeneTex, Irvine, CA, USA) at a dilution of 1:1,000 for 1 h at room temperature. The excess primary antibodies were washed off with TBS-T, and the membranes were treated with a secondary antibody, anti-mouse, or anti-rabbit IgG (H + L) antibody-conjugated horseradish peroxidase (HRP) (Thermo Fisher Scientific), each at a dilution of 1:10,000, for 30 minutes at room temperature. The bound conjugated antibody was detected using Immobiron Western Chemiluminescent HRP Substrate (Merck) by ImageQuant LAS4000 (Cytiva).

### Cryo-EM grid preparation and data collection

Samples were preincubated with a protein concentration of 250 µg/mL at 37°C for 1 h, and 3 µL of samples was applied to freshly glow-discharged Quantifoil R1.2/1.3 Cu 300 mesh grids (Quantifoil Micro Tools GmbH, Jena, Germany). The grids were flash frozen in liquid ethane using a Vitrobot Mark IV (Thermo Fisher Scientific) at 18°C, 100% humidity, blotting time of 5 s, and blotting force 5 setting. Micrographs were collected on a Krios G4 (Thermo Fisher Scientific) operated at 300 kV with a K3 direct electron detector (Gatan, Pleasanton, CA, USA) at a nominal magnification of 130,000 (0.67 Å per physical pixel) using a GIF-Biocontinuum energy filter (Gatan) with a 10 eV slit width. Each micrograph was collected with a total exposure of 0.9 s and a total dose of 52.85 e/Å2. A total of 3,500 micrographs were collected at a nominal defocus range of −0.5 to −3.0 µm using EPU software (Thermo Fisher Scientific).

### Cryo-EM image processing

All image processing steps were performed using cryoSPARC (v.4.2.1) (76). Motion correction was performed using Patch motion correction. CTF for each micrograph was estimated by Patch CTF estimation. Particles were autopicked by a blob picker using all micrographs, and 1,448,075 particles were extracted using binning state (3.57 Å/pixel). Two rounds of 2D classification (class = 150, iteration = 30, batch size = 200) were then performed. An initial model was reconstituted by ab initio reconstruction using selected particles. Heterogeneous refinement was performed with two classes. After re-extracting using the particles in 0.89 Å/pixel (Nyquist Resolution 1.78 Å), 31,127 particles were selected by 2D classification. Finally, 3D reconstruction was performed by non-uniform refinement, and a 3.49 Å map was obtained. The reported resolutions are based on the gold-standard Fourier shell correlation (FSC) curves (FSC = 0.143) criterion. Figures were prepared with UCSF Chimera (v.1.17) (76).

### Mice

Female C57BL/6 mice aged 6–8 weeks were purchased from CLEA Japan (CLEA Japan Inc., Tokyo, Japan) and maintained in a BSL-2 and BSL-3 laboratory at the International Institute for Zoonosis Control, Hokkaido University, under standard laboratory conditions (temperature of 22°C ± 2°C, humidity of 50% ± 10%, and a 12 h/12 h light/dark cycle). All mice were given food pellets (CE-2; CLEA Japan, Sapporo, Japan) and water *ad libitum*.

### Vaccination and serum collection

In each vaccination group, 10 µg of S antigen formulated with or without 10 µg of ARNAX in 100 µL of PBS was injected subcutaneously once or twice at 2 week intervals. As a representative with licensed adjuvants, 10 µg of S antigen formulated with 25 µL of InjectAlum (Thermo Fisher Scientific) or AddaVax (InvivoGen, San Diego, CA, USA) was injected intramuscularly. XBB.1.5 mRNA vaccine (BNT162b2, Pfizer-BioNTech) was diluted fivefold with PBS, and 50 µL (1 µg RNA) was injected intramuscularly into mice. PBS was injected subcutaneously to mice as a non-vaccination control. In addition, 10 µg of poly I:C (Cytiva) plus 10 µg of S antigen was injected subcutaneously to mice as a positive control to measure cytokines and chemokines in serum. The blood samples for assessment of antibody titer were collected from immunized mice 3–24 h after the first dose of immunization for cytokine and chemokine assays and a few days before the second dose immunization and virus challenge for antibody measurement. Those were centrifuged at 2,000 × *g* for 10 minutes after incubation at 4°C for 1 h to separate sera. The sera were stored at –80°C until use.

### Cytokine and chemokine analysis

The concentration of cytokines and chemokines in serum samples was measured by LEGENDplex Mouse Anti-Virus Response Panel (BioLegend, San Diego, CA, USA) according to the manufacturer’s instructions. The data were obtained using BD FACS Melody and were analyzed using LEGENDplex data analysis software.

### Flow cytometry analysis

Spleens and inguinal lymph nodes were collected from immunized mice 10 days after the first vaccination or 4 days after the second vaccination. The lymph nodes from all mice in each group were combined and examined as a single sample. For detection of Tfh T cells and GC B cells, the following antibodies for staining cell surface markers were used: CD4 (RM4-5), CD3 (17A2), CD44 (IM7), CXCR5 (L138D7), PD-1 (29F.1A12), CD45 (30-F11), CD19 (6D5), B220 (RA3-6B2), GL7 (GL7), and Fas (SA36H8).

For intracellular cytokine staining, single-cell suspensions from the spleens and lymph nodes were cultured in Roswell Park Memorial Institute (RPMI) 1640 medium containing 100 U/mL of penicillin and 100 µg/mL of streptomycin, 20 µg/mL of gentamicin, 50 µM of 2-mercaptoethanol, 10% FCS, 10 U/mL of human IL-2 recombinant (Sigma-Aldrich), and 2 µL/mL of BD GolgiPlug (BD Bioscience, San Jose, CA, USA), with or without 2 µg/mL of SARS-CoV-2 S peptides for 12 h. The SARS-CoV-2 S peptides used were either chain A or SARS-CoV-2 spike glycoprotein (539–546) (MBL International Corporation, Woburn, WA, USA), which is H-2K^b^ restricted or a SARS-CoV-2 S1 scanning pool of 166 peptides (Mabtech, AB, Sweden). As a positive control, ionomycin (Sigma-Aldrich) and phorbol 12-myristate 13-acetate (Sigma-Aldrich) were used to stimulate T cells non-specifically.

After 12 h incubation, the cells were washed with PBS containing 2.5% FCS (FACS buffer) and then stained with antibodies for cell surface markers CD8α (53–6.7), CD4 (RM4-5), CD3 (17A2), CD44 (IM7), and CD62L (MEL-14) and Fixable Viability Dye eFluor 506 (Thermo Fisher Scientific), in the presence of Fc blocking antibody (anti-CD16/CD32, BioLegend) for 40 minutes at 4°C in the dark. The cells were then permeabilized using the BD Cytofix/Cytoperm kit (BD Bioscience) according to the manufacturer’s instructions followed by staining with antibodies for intracellular cytokines, anti-IL-2 (JES6-5H4), IFN-γ (XMG1.2), and TNF-α (MP6-XT22), in the presence of Fc blocking antibody for 40 minutes at 4°C in the dark. All antibodies for staining of cell surface markers and intracellular cytokines were purchased from BioLegend. The flow cytometry data were acquired by BD LSRFortessa and analyzed using FlowJo software (v.10, BD Bioscience).

### Virus challenge and titration

Two weeks after the last vaccination, mice were inoculated intranasally with 1 × 10^5^ PFU of mouse-adapted SARS-CoV-2 MA-P10 per mouse under anesthesia with isoflurane. Mice at the time of virus challenge were 11–12 weeks of age. Body weight was monitored daily after infection. Whole lungs were harvested 3 days post-infection and homogenized in 1.5 mL of RPMI 1640 medium supplemented with 100 U/mL penicillin, 100 µg/mL streptomycin, and 20 µg/mL gentamicin, centrifuged at 800 × *g* for 15 minutes to obtain the supernatant, and stored at −80°C until use.

Virus titers of lung samples were determined by plaque assay modified from a previous method (3). Briefly, VeroE6/TMPRSS2 or VeroE6/TMPRSS2-ACE2 cells were seeded into six-well plates at a density of 1.0 × 10^6^ cells per well and incubated for 1 day at 37°C in 5% CO_2_ to form cell monolayers. The supernatants from lung samples were serially diluted 10-fold from 10^−1^ to 10^−6^ in DMEM supplemented with 100 U/mL of penicillin and 100 µg/mL of streptomycin, and 125 µL of dilutants was added to each well. After incubation for 1 h at 37°C, 3 mL of warmed Leiboviz’s L-15 medium (Thermo Fisher Scientific) containing 100 U/mL penicillin, 100 µg/mL streptomycin, and 0.9% Noble agar (Becton Dickinson, USA) was overlaid on the cell monolayer in each well. Plaques formed in the cell monolayers were counted after incubation for 3 days at 37°C in 5% CO_2_, and PFU per lung was calculated.

### Titration of antibodies

Serum samples for antibody titer assay were inactivated by incubation at 56°C for 30 minutes and then diluted 10-fold with PBS. Antigen-specific antibody titers were measured by ELISA. Ninety-six-well plates (R&D Systems, Minneapolis, MN, USA) were coated with 100 µg per well of S antigen and incubated at 37°C for 2 h. The plates were washed with PBS containing 0.05% Tween-20 (PBST) and blocked with PBS containing 5% FCS at 37°C for 2 h. Serum samples were serially diluted threefold in PBS containing 5% FCS and 0.05% Tween 20. Subsequently, the diluted serum samples were added to the plate and reacted with the coated antigen at room temperature for 1 h. After washing with PBST, HRP-conjugated anti-mouse IgG (H + L) antibody (Southern Biotech, Alabama, USA) diluted at 1:6,000 was added to the plate and incubated at room temperature for 30 minutes. The plates were washed and reacted with OPD Peroxidase substrate (Sigma-Aldrich) for 15 minutes. After stopping the reaction with 3N H_2_SO_4_, the absorbance at 490 nm was measured. S antigen-specific antibody titers were defined as the highest serum dilution that yielded an absorbance greater than the negative control.

Neutralizing antibody titers in mice were evaluated by a PRNT. Serum samples were serially diluted twofold in the range of 20–1,280 dilutions in DMEM containing 100 U/mL of penicillin and 100 µg/mL of streptomycin. Each 50 µL of diluted sample was mixed with 100 PFU of an equal volume of SARS-CoV-2 WK-521 (ancestral strain) and incubated at 37°C for 1 h. Subsequently, 100 µL of the serum-virus mixtures was inoculated to VeroE6/TMPRSS2 or VeroE6/TMPRSS2-ACE2 cells, forming a monolayer in the well of six-well plates. Following incubation at 37°C in 5% CO_2_ for 1 h, 3 mL of warmed Leiboviz’s L-15 medium containing 100 U/mL penicillin, 100 µg/mL streptomycin, and 0.9% Noble agar was overlaid on the cell monolayers in each well. After incubation at 37°C in 5% CO_2_ for 3 days, the number of plaques on the cell monolayers was counted, and virus neutralizing antibody titers were defined as the highest serum dilutions at which the plaque count was reduced by 50%.

### Histopathological analysis

Mouse lung samples (left robe) were harvested 3 days after virus challenge and fixed with MildformR 10N (Fujifilm Wako, Osaka, Japan) for 10 days. All samples were embedded in paraffin and sectioned, and the sections were stained with H&E. Images of stained tissue samples were captured with a NanoZoomer scanner (HAMAMATSU Photonics, Hamamatsu, Japan) and analyzed by NDPview2 (HAMAMATSU Photonics).

### Statistical analysis

GraphPad Prism 10 (GraphPad Software, Boston, MA, USA) was used to perform statistical analysis. To detect differences between groups, data were analyzed by one-way or two-way analysis of variance (ANOVA), followed by Tukey’s test or Brown-Forsythe ANOVA followed by Dunnett’s T3 test.

## Data Availability

The protein structure data obtained in this study have been deposited in the Electron Microscopy Data Bank (EMDB) under code EMD-63071.

## SUPPLEMENTAL MATERIAL

Supplemental material jvi.02290-24-s0001.pdf Figures S1 to S5 and Table S1.

ASM does not own the copyrights to Supplemental Material that may be linked to, or accessed through, an article. The authors have granted ASM a non-exclusive, world-wide license to publish the Supplemental Material files. Please contact the corresponding author directly for reuse.
