# Supplementary material for: ARNAX is an ideal adjuvant for COVID-19 vaccines to enhance antigen-specific CD4+ and CD8+ T-cell responses and neutralizing antibody induction
Source: J Virol. 2025 Apr 15;99(5):e02290-24. doi: 10.1128/jvi.02290-24 (PMC12090777; doi:10.1128/jvi.02290-24)
Supplement: Supplemental material — Figures S1 to S5 and Table S1. [file jvi.02290-24-s0001.pdf]

Supplemental Information

ARNAX is an ideal adjuvant for COVID-19 vaccines to enhance antigen-specific $CD4^+$ and $CD8^+$ T cell responses and neutralizing antibody induction

Tomomi Kawakita [1,2,3], Toshiki Sekiya [2,3,4,5], Yayoi Kameda [6], Naoki Nomura [3,7], Marumi Ohno [2,3,8], Chimuka Handabile [2,3], Akari Yamaya [9], Hideo Fukuhara [2, 10], Yuki Anraku [11], Shunsuke Kita [2,11], Shinsuke Toba [12, 13], Hirotake Tsukamoto [14], Tomohiro Sawa [15], Hiroyuki Oshiumi [16], Yasushi Itoh [17], Katsumi Maenaka [2,10,11,18], Akihiko Sato [2, 12, 13], Hirofumi Sawa [2, 4, 8, 13], Yasuhiko Suzuki [2,6], Lorena E Brown [4,5], David C Jackson [4,5], Hiroshi Kida [1,2,3,4], Misako Matsumoto [1,9,19], Tsukasa Seya [1,9,19,*], Masashi Shingai [1,2,3,4*].

[1] Division of Vaccine Immunology, Hokkaido University International Institute for Zoonosis Control, Sapporo, Japan
[2] Institute for Vaccine Research and Development (HU-IVReD), Hokkaido University, Sapporo, Japan
[3] Division of Biologics Development, International Institute for Zoonosis Control, Hokkaido University, Sapporo, Japan
[4] International Collaboration Unit, International Institute for Zoonosis Control, Hokkaido University, Sapporo, Japan
[5] The Department of Microbiology and Immunology, The University of Melbourne at the Peter Doherty Institute for Infection and Immunity, Melbourne, Australia.
[6] Division of Bioresources, International Institute for Zoonosis Control, Hokkaido University, Sapporo, Japan
[7] Division of International Research Promotion, International Institute for Zoonosis Control, Hokkaido University, Sapporo, Japan
[8] One Health Research Center, Hokkaido University, Sapporo, Japan
[9] Nebuta Research Institute for Life Sciences, Aomori University, Aomori, Japan
[10] Division of Pathogen Structure, International Institute for Zoonosis Control, Hokkaido University, Sapporo, Japan.
[11] Laboratory of Biomolecular Science, and Center for Research and Education on Drug Discovery, Faculty of Pharmaceutical Sciences, Hokkaido University; Sapporo, Hokkaido, Japan.
[12] Shionogi Pharmaceutical Research Center, Shionogi & Company, Limited, Toyonaka, Japan
[13] Division of Molecular Pathobiology, International Institute for Zoonosis Control, Hokkaido University, Sapporo, Japan
[14] Division of Clinical Immunology and Cancer Immunotherapy, Center for Cancer Immunotherapy and Immunobiology, Graduate School of Medicine, Kyoto University, Kyoto, Japan.
[15] Department of Microbiology, Graduate School of Medical Sciences, Kumamoto University, Kumamoto, Japan.
[16] Department of Immunology, Graduate School of Medical Sciences, Faculty of Life Sciences, Kumamoto University, Kumamoto, Japan.

[17] Division of Pathogenesis and Disease Regulation, Department of Pathology, Shiga University of Medical Science, Otsu, Japan.
[18] Global Station for Biosurfaces and Drug Discovery, Hokkaido University; Sapporo, Hokkaido, Japan.
[19] Department of Vaccine Immunology, Hokkaido University Graduate School of Medicine, Sapporo, Japan

*Corresponding authors: Tsukasa Seya and Masashi Shingai
International Institute for Zoonosis Control, Hokkaido University, Kita-20 Nishi-10, Kita-ku, Sapporo 001-0020, Hokkaido, Japan
Tel: +81-11-706-9494; FAX: +81-11-706-9548
E-mail: seya-tu@pop.med.hokudai.ac.jp and shingaim@czc.hokudai.ac.jp

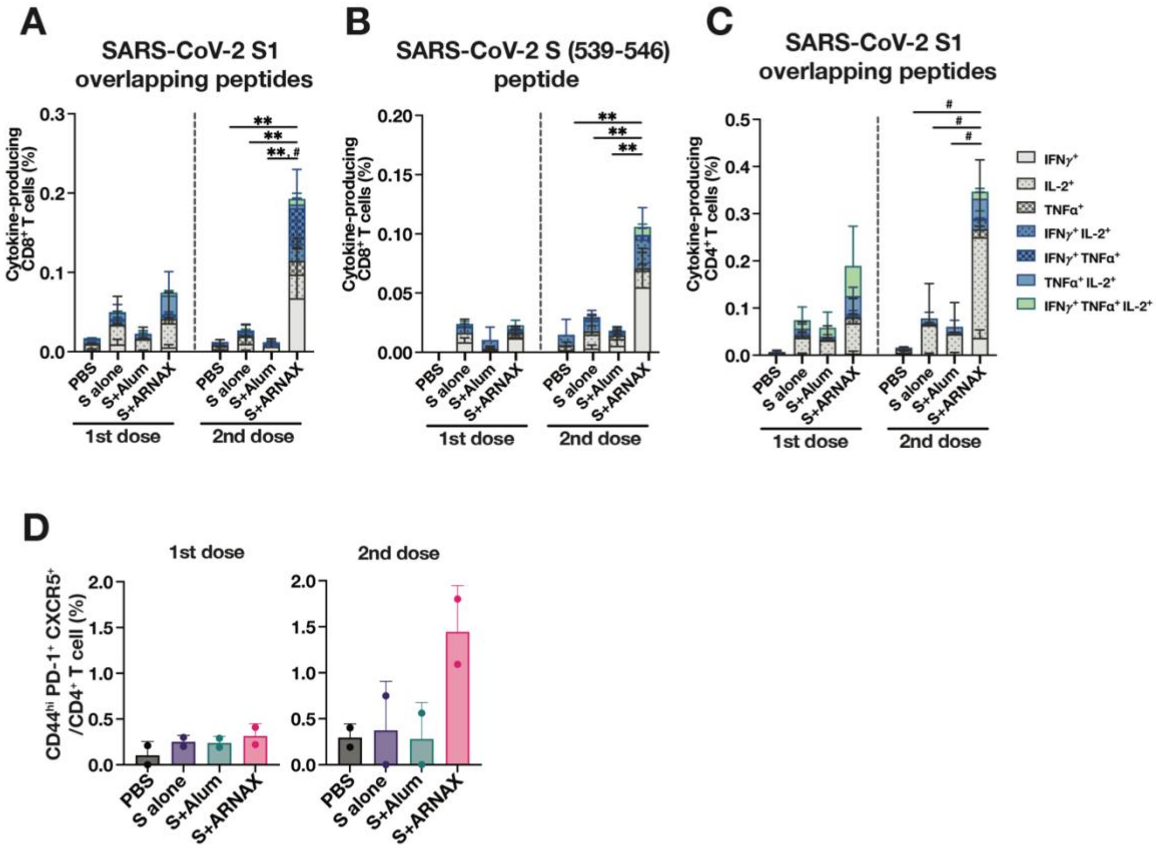

Fig S1. Analysis of cytokine producing T cells

Mice were immunized with each vaccine (n = 7-10). Ten days after the first dose or 4 days after the second dose of vaccine, lymph nodes were harvested. The lymph nodes from 3-4 mice for each experiment were pooled within the vaccine group as a single sample. The pooled lymph node cells were re-stimulated with overlapping peptides (A, C) or AA 539-546 peptide of S protein (B). The results of 3 independent experiments are combined and shown in A, B, and C. Percentages of cytokine-producing $CD4^+$ and $CD8^+$ T cells (IFN-γ, IL-2, and TNF-α) were analyzed by intracellular cytokine staining using flow cytometry. Individual cytokine patterns are represented by the following: light gray for IFN-γ^+, light gray with small dots for IL-2^+, light gray with large dots for TNF-α^+, light blue with small dot for IFN-γ^+ IL-2^+, light blue with large dot for IFN-γ^+ TNF-α^+, light blue for TNF-α^+ IL-2^+, green for IFN-γ^+ TNF-α^+ IL-2^+. Each error bar represents mean ± SD. Significance is calculated for total cytokine-producing cells and multiple cytokine-producing cells (colored bars) using Two-way ANOVA with Tukey's multiple comparisons test. For total cytokine-producing cells: $^{**}P<0.01$, multiple cytokine-producing cells: $^{\#}P<0.05$. The percentage of Tfh, defined as $CD44^{hi}$, PD-1^+, and $CXCR5^+$, in $CD4^+$ T cells in lymph nodes was analyzed (D). The results of 2 independent experiments are combined and shown in D.

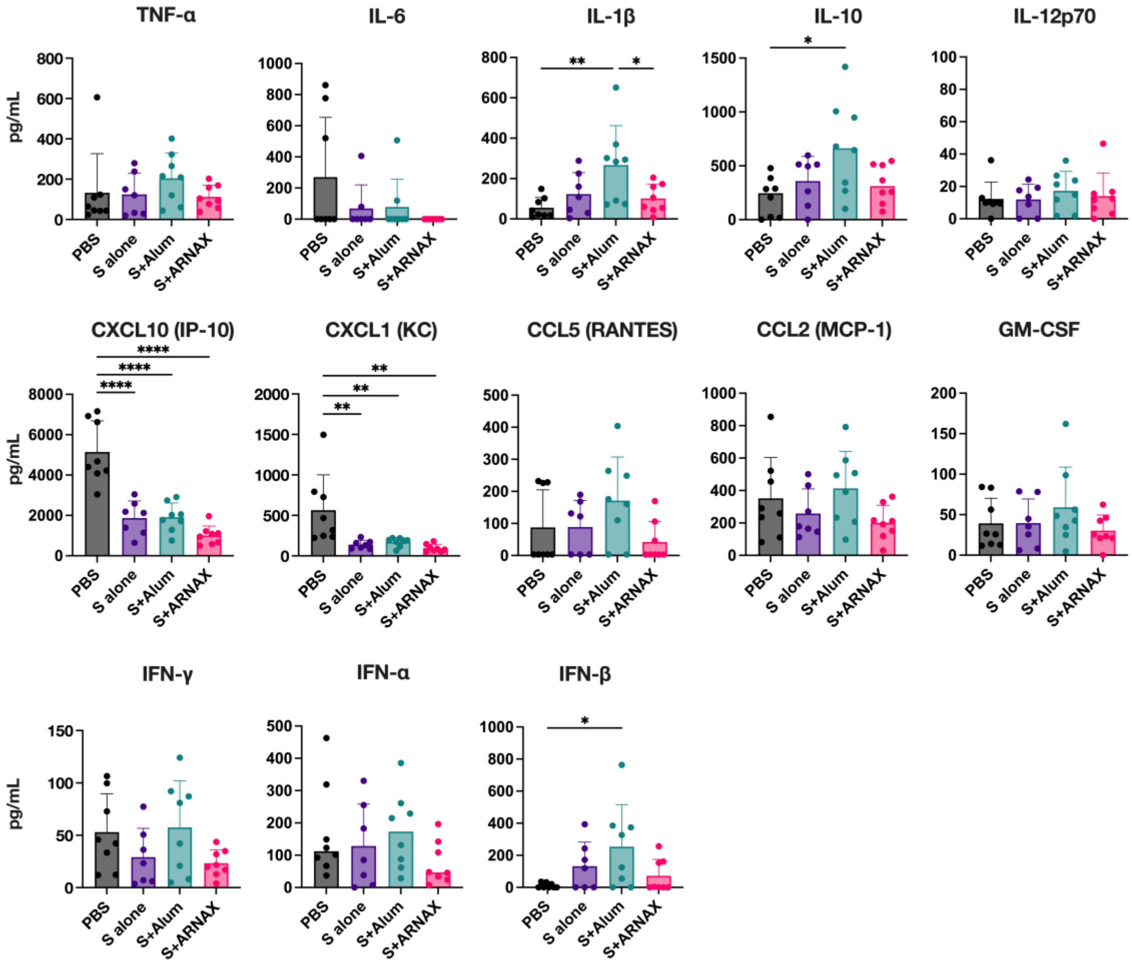

Fig. S2. Cytokine and chemokine levels in the sera at 3 days post-infection

Serum samples collected at 3 dpi were analyzed in a bead-based immunoassay to measure concentrations of cytokines and chemokines as indicated. Individual samples are shown as dots, horizontal lines indicate the median (n = 8). Significance was calculated using One-way ANOVA with Tukey's multiple comparisons test. *P<0.05, **P<0.01, ****P<0.0001.

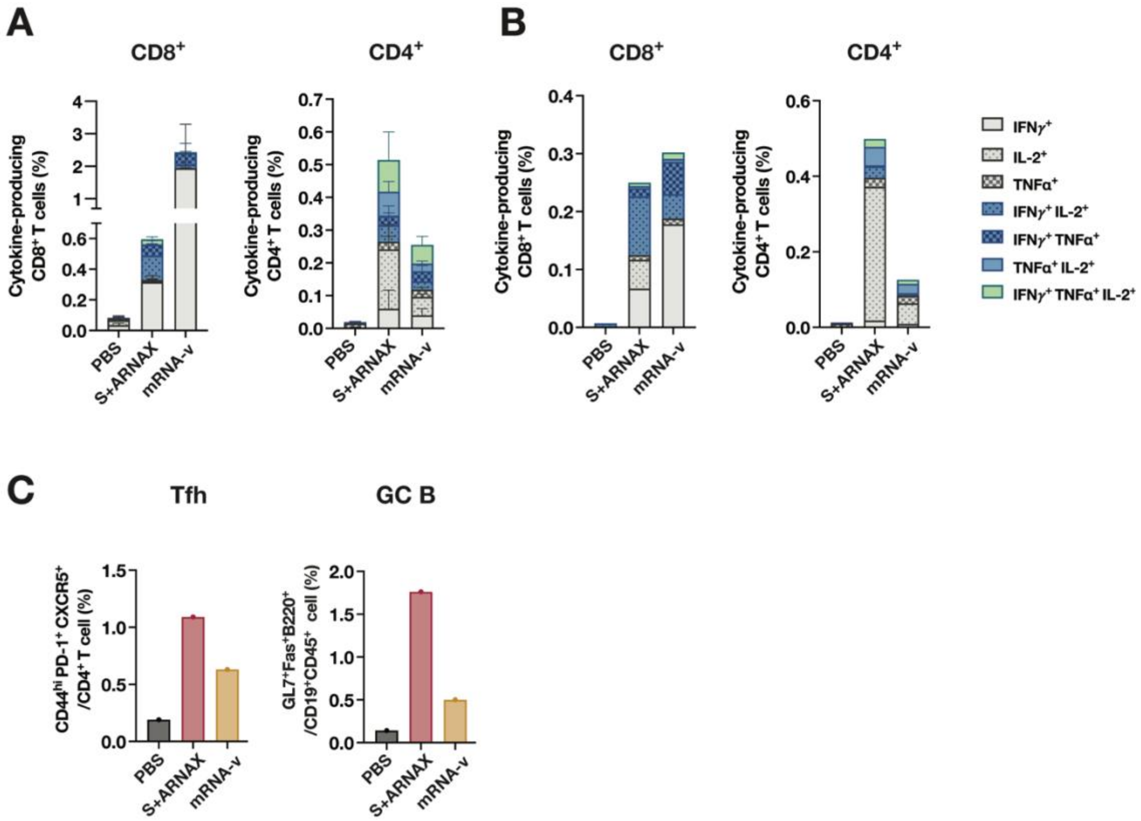

Fig S3. Comparison of T cell responses between S plus ARNAX and mRNA vaccines

Mice were immunized with each vaccine (n = 3). Four days after the second dose of vaccine, spleen and lymph nodes were harvested. The lymph nodes cells from 3-4 mice for each experiment were pooled within the vaccine group as a single sample. The splenocytes (A) and pooled lymph node cells (B) were re-stimulated with overlapping peptides of S protein. Percentages of cytokine-producing $CD4^+$ and $CD8^+$ T cells (IFN-γ, IL-2, and TNF-α) were analyzed by intracellular cytokine staining using flow cytometry. Individual cytokine patterns are represented by the following: light gray for IFN-γ^+, light gray with small dots for IL-2^+, light gray with large dots for TNF-α^+, light blue with small dot for IFN-γ^+ IL-2^+, light blue with large dot for IFN-γ^+ TNF-α^+, light blue for TNF-α^+ IL-2^+, green for IFN-γ^+ TNF-α^+ IL-2^+. Each error bar represents mean ± SD for splenocytes. The percentage of Tfh (defined as $CD44^{hi}$, PD-1^+, and $CXCR5^+$) in $CD4^+$ T cells and GCB (defined as $GL7^+$, Fas^+, $B220^+$) cells in $CD19^+$ $CD45^+$ B cells in lymph nodes was analyzed (C). Results are representative of at least two independent experiments.

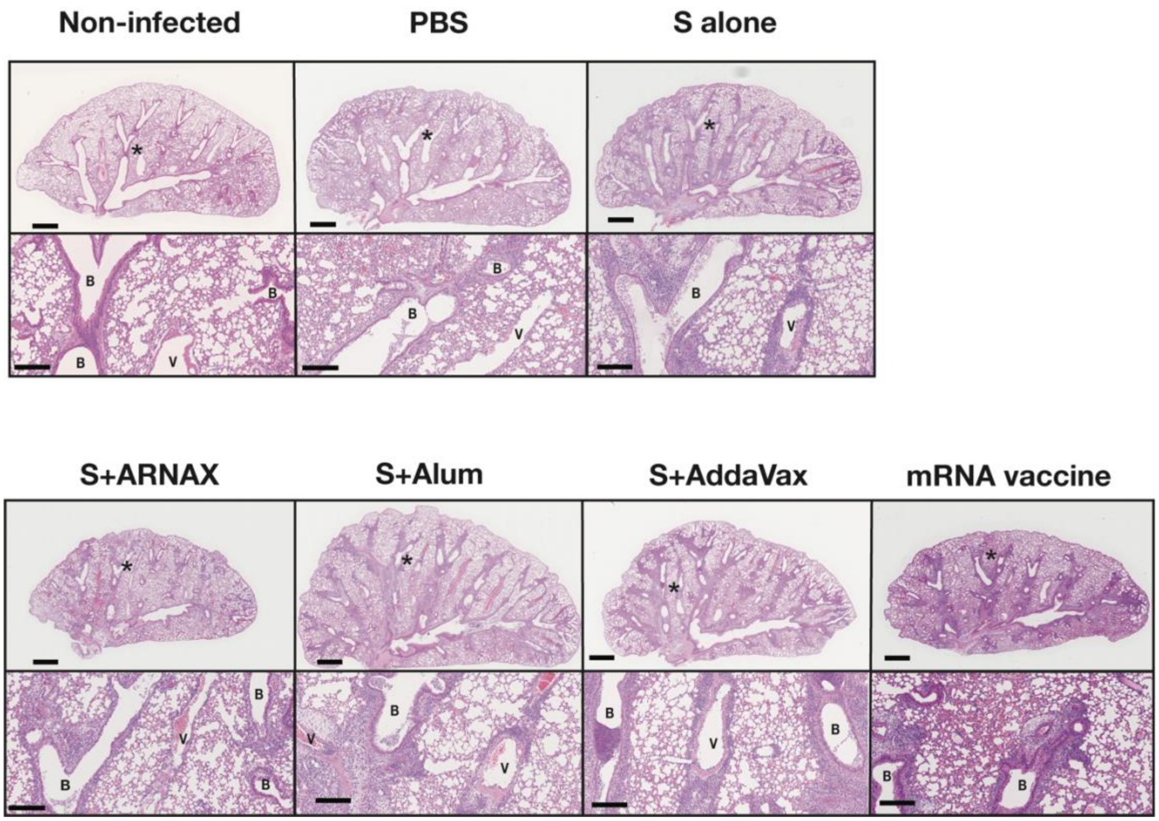

Fig S4. Histopathology of lung section at 3 dpi

Mice were challenged with 1 × 10^5 PFU of SARS-CoV-2 mouse-adapted strain 2 weeks after the second dose. The lung tissues were harvested at 3 dpi and stained with H&E. Representative pictures of each group (n = 5) were shown. The asterisk of upper panel is indicated indicates an enlarged area (bottom panel). The scale bars indicated 1mm (upper panel) and 250 µm. Bronchiole and vessel are marked as B and V respectively.

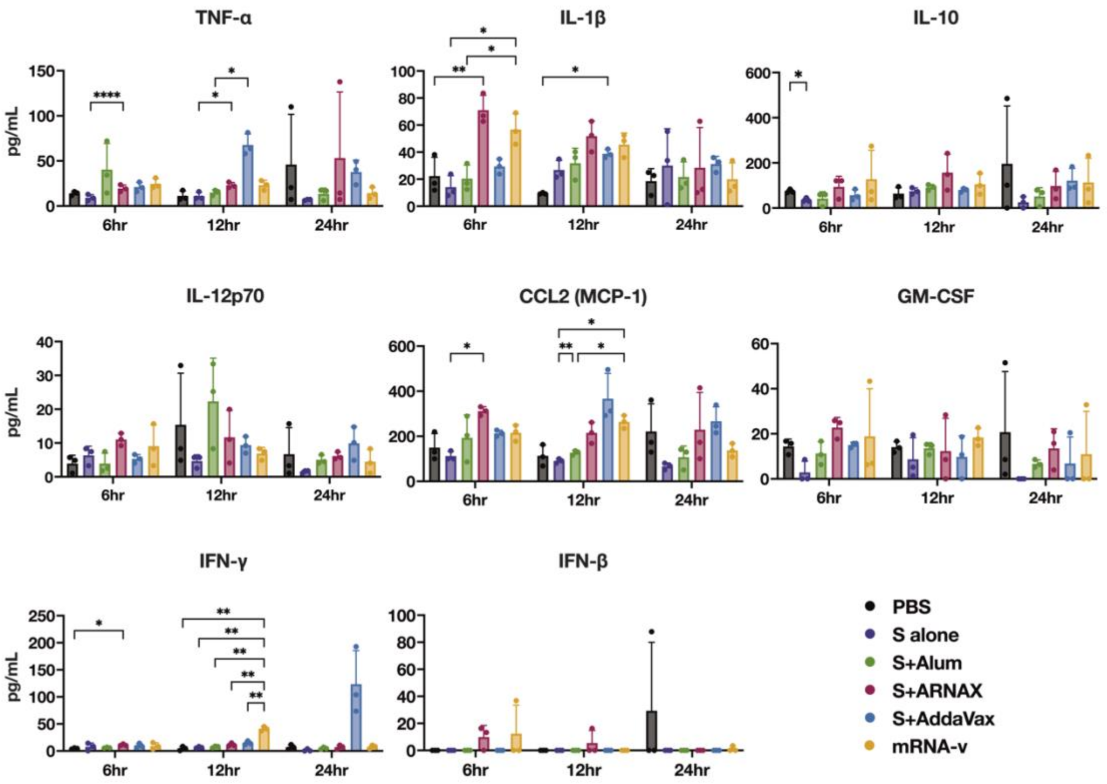

Fig S5. Cytokine and chemokine levels 6-24 hours after vaccination

Sera were collected from immunized mice at 6-24 hours after the first dose of vaccine, and concentrations of the indicated cytokines and chemokines were measured using a bead-based immunoassay (n = 3). Individual samples are shown as dots, horizontal lines indicate the median. Significance was calculated using One-way ANOVA with Tukey's multiple comparisons test. *P<0.05, **P<0.01, ****P<0.0001.

Table S1 Cryo-EM data collection, refinement, and validation statistics

	EMD-63071 spike protein
EMDB entry	EMD-63071
Data collection and processing	
Magnification	130,000
Microscope	Krios G4
Voltage (kV)	300
Detector	Gatan K3
Energy filter	Gatan BioContinuum, 10 eV slit
Electric exposure (e-/Å)	52.85
Deforcus range (μm)	−0.5 to −3.0
Pixel size (Å)	0.67
Data Processing Program	CryoSPARC (v.4.2.1)
Movies	3,500
Initial / Final particle images (no.)	14,448,075 / 31,127
Symmetry imposed	C1
Map resolution (Å)	3.67
FSC threshold	0.143
